# Development of a new efficient and economical magnetic sorbent silicone surfactant-based activated carbon for the removal of chloro- and nitro-group phenolic compounds from contaminated water samples†

**DOI:** 10.1039/c9ra07151b

**Published:** 2019-11-13

**Authors:** K. Gopal, N. I. Mohd, M. Raoov, F. B. M. Suah, N. Yahaya, N. N. M. Zain

**Affiliations:** Integrative Medicine Cluster, Advanced Medical and Dental Institute, Universiti Sains Malaysia 13200 Kepala Batas Penang Malaysia nurnadhirah@usm.my; Department of Chemistry, Faculty of Science, Universiti Malaya Kuala Lumpur 50603 Malaysia; School of Chemical Sciences, Universiti Sains Malaysia Pulau Pinang 11800 Malaysia

## Abstract

In this study, activated carbon (AC) coated with a green silicone surfactant (SS) was further incorporated with magnetite particles (Fe_3_O_4_) *via* a co-precipitation method to enhance the separation of the newly designed magnetic AC–SS (Fe_3_O_4_@AC–SS) in a magnetic field. The properties of this magnetic adsorbent were characterized *via* Fourier transform-infrared spectroscopy (FT-IR), thermogravimetric analysis (TGA), X-ray diffraction (XRD), and transmission electron microscopy (TEM). The adsorption characteristics of the Fe_3_O_4_@AC–SS adsorbent were examined using 2,4-nitrophenol and 2,4-dichlorophenol as adsorbates. Experiments were performed to investigate the adsorption kinetics, isotherms, thermodynamics as well as the effects of adsorption dosage and solution pH on the removal of both analytes. The kinetic data were well-fitted by the pseudo-second order model and the Freundlich model best described the adsorption isotherm for both analytes. The maximum adsorption capabilities for 2,4-dinitrophenol and 2,4-dichlorophenol reached 43 and 98 mg g^−1^, respectively. The analysis was further validated using real industrial effluent, and a removal efficiency of 62.2–98.1% and relative standard deviation value less than 7.2% were attained for both analytes. Thus, the multifunctional adsorbent has potential to function as an adsorbent for the fast, convenient, economical and highly efficient removal of pollutants from wastewater, which is significant for the purification of natural water and industrial effluent.

## Abstract 2

In this study, activated carbon (AC) coated with a green silicone surfactant (SS) was further incorporated with magnetite particles (Fe_3_O_4_) to enhance the separation of the newly designed magnetic AC–SS (Fe_3_O_4_@AC–SS) in a magnetic field.

## Graphical abstract

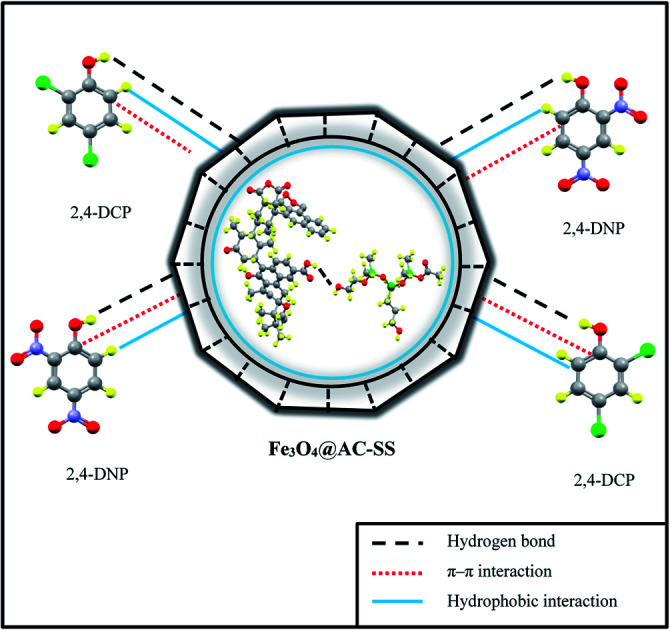

## 1 Introduction

Phenolic compounds and their derivatives have become emerging concerns as they pose a high-level threat to human health and the ecosystem due to their widespread use in the petrochemical, pharmaceutical, pesticide, plastic and paper industries.^1^ They contain a hydroxyl group bonded directly to a benzene ring, and hence these phenolic compounds possess a stable conjugated system that leads to biological accumulation and poor biodegradation.^2^ Phenolic compounds are also scientifically proven to induce carcinogenicity, genotoxicity, immunotoxicity and physiological effects.^3^ Even minor exposure towards phenolic compounds can result in many health-related problems such as cancer, nausea, vomiting, paralysis, smoky colored urine, diarrhea, severe skin burns and mouth sores.^4^ Their presence in the ecosystem is related to the discharge of polluted effluent, and therefore the treatment of such sewage prior to discharge is a considerable challenge due to these toxic compounds and their contribution to health problems.^5,6^ Stringent US Environmental Protection Agency (EPA) regulations have listed phenolic compounds as primary pollutants^7^ and set the limit of phenolic compounds content in wastewater at 1 mg L^−1^.^8^ The adverse effects of these compounds have been highlighted by the Malaysian Department of Environment (DOE) by stipulating that the content level of phenolic compounds in wastewater should not to exceed 0.001 mg L^−1^.^9^ In this study, due to the high level of persistence and environmental waste discharge, we examine 2,4-dichlorophenol (2,4-DCP) and 2,4-dinitrophenol (2,4-DNP) phenolic compounds.^6^

Ideally, the removal processes must be simple, effective and inexpensive. Several methods have been applied to remove phenolic compounds in wastewaters. These processes include microbial degradation,^7^ membrane,^10^ chemical oxidation,^11^ electrochemical degradation,^12^ photocatalytic degradation,^13^ and solvent-based extraction.^14^ However, despite their advantages, these techniques have remarkable drawbacks such as, large waste production, high use of current and chemicals during the removal process, low efficiency in removing pollutants, regeneration of toxic byproducts, residual effects and difficult operational techniques.^15^ Thus, the among various physiochemical processes, adsorption has gained considerable attention for treating phenolic compounds from wastewater due to its low cost, flexibility and simplicity of design, ease of operation and insensitivity to toxic pollutants.^16^ Recently, the application of magnetic particle technology has received considerable attention to solve environmental problems. Magnetic particles can be used to adsorb contaminants from aqueous effluents, then after adsorption, the magnetic particles can be separated from the medium *via* simple magnetic field application. Nevertheless, magnetic particles have the drawbacks of small surface area and small adsorption capacity, which restrict their wide application. In this work, high surface area and high adsorption capacity magnetic particles based on activated carbon (AC) were prepared using a simple method, which served as an adsorbent to eliminate pollutants from aqueous effluents.

AC is an effective and inexpensive adsorbent widely used in various industries to purify water.^17^ It removes phenolic compounds effectively due to its high adsorption capacity, porosity, high surface area and reactivity.^18^ However, in commercial AC, these functional groups cover only a small portion of the carbon surface, and thus an increase in the quantity of these groups may enhance the capacity of AC to adsorb organic compounds. Since phenolic compounds are low polar and high polar,^19^ the particles can be combined with the amphipathic structure of surfactants with a hydrophobic tail and hydrophilic head. Accordingly, the surfactant can modify the characteristics of the solid AC surface. Thus, OFX 0309 fluid, which is a non-ionic silicone surfactant (SS) based on polyethylene glycol, was used in surface modification of AC. This surfactant is non-toxic, odorless, colorless, non-irritating, and does not evaporate easily.^20^ It is also considered inert since it does not react with other materials.^21^ Besides, surfactants have become an important and growing class of raw materials used in the petroleum, medicine, cosmetic, food, and pharmaceutical industries.^22^ Its biocompatibility and safety to both humans and the environment have been proven since in the past.^23,24^

This study aimed to enhance the capacity of commercial AC in adsorbing 2,4-DNP and 2,4-DCP from wastewater using magnetic activated carbon impregnated with a silicone surfactant (Fe_3_O_4_@AC–SS), which is absorbent and cost effective. The surface of AC was modified by impregnating it with non-ionic silicone surfactant and by coating the material with magnetic nanoparticles. The development of this unique adsorbent can increase the viability of the adsorbent in removing chloro- and nitro-group phenolic compounds simultaneously in a more efficient way compared to the existing developed adsorbents. Besides, the developed adsorbent Fe_3_O_4_@AC–SS can also be used as an alternative adsorbent in the removal of other existing pollutants. Various adsorption parameters were examined, and the data obtained were modeled and optimized to provide useful information for environmental scientists/engineers in designing effective and low-cost wastewater treatment materials. The surface properties of the synthesized material were assessed, in addition to determining how these properties correlate with the synthesized material to be able to adsorb the selected phenolic compounds.

## 2 Materials and methods

### 2.1 Chemical and reagents

Ferrous chloride tetrahydrate (FeCl_2_·4H_2_O), ferric chloride hexahydrate (FeCl_3_·6H_2_O) and sodium hydroxide (NaOH) were purchased from R&M Chemicals (Edmonton, Canada). Ethanol absolute (denatured) was obtained from HmbG Chemicals (Cologne, Germany). Non-ionic silicone surfactant (OFX 0309 fluid) 3-(3-hydroxypropyl-heptatrimethylxyloxane), and 5500 g mol^−1^ of Xiameter was obtained from Ingredient Plus, Selangor, Malaysia. Ammonia solution (25%) (NH_4_OH_2_) was purchased from Merck, Darmstadt, Germany. Hydrochloric acid (HCl) and granulated activated carbon were purchased from Friedemann Schmidt Chemicals (Parkwood, Australia). The phenolic compounds, 2,4-DNP and 2,4-DCP, were obtained from Sigma Aldrich (St Louis, Missouri, US). All reagents and chemicals were used without further purification.

### 2.2 Instrumentation

A PerkinElmer Lambda 25 UV spectrophotometer (Waltham, Massachusetts, US) was used to analyze both 2,4-DCP (*λ*_max_: 284.6 nm) and 2,4-DNP (*λ*_max_: 358.5 nm). An orbital shaker (Orbitron, INFORS HT) from Bottmingen, Switzerland was used to shake the samples during the adsorption process. The pH of the solution was adjusted using a pH meter from Hanna Instruments (Rhode Island, USA).

### 2.3 Synthesis of magnetic particles

In this research, Fe_3_O_4_ particles were synthesized using a conventional co-precipitation method with slight modification.^25^ FeCl_2_·4H_2_O and FeCl_3_·6H_2_O (1 : 2 molar ratio) were dissolved in 75 mL of deionized water. The resulting clear orange solution was stirred vigorously at 85 °C under a nitrogen stream. After 30 min, 10 mL of NH_4_OH_2_ solution (25%) was added to the solution mixture. The orange iron solution turned black upon the addition of NH_4_OH_2_, which indicated the formation of magnetic particles. The formed precipitate was stirred and heated to 90 °C for 1 h. The formed precipitate was collected using an external magnet. Next, the precipitate was washed with deionized water and ethanol several times to discard the unreacted chemicals. The final product was oven-dried at 80 °C for 24 h, and crushed and sieved for further use.

### 2.4 Modification of magnetic activated carbon impregnated with silicone surfactant

The final material of Fe_3_O_4_@AC–SS was synthesized in two steps. (1) Modification of AC surface with SS and (2) magnetization of AC impregnated with SS based on a previously developed method by Mohd *et al.*^26^ with slight modification. Initially, 100 mg of AC was mixed with 20% w/v OFX 0309 SS in ethanol to form the AC–SS material. The AC–SS mixture was stirred for 24 h at room temperature. Next, the AC–SS mixture was filtered and oven-dried for 24 h. In the second step, 100 mg of the synthesized AC–SS mixture was added to FeCl_2_·4H_2_O (1.4576 g) and FeCl_3_·6H_2_O (3.7306 g). Then distilled water was added to this mixture and stirred at 85 °C at high speed under a nitrogen stream. After 30 min, 10 mL 25% NH_4_OH_2_ was added to the above mixture, and it was left to stir for another hour. Finally, the synthesized Fe_3_O_4_@AC–SS was washed with deionized water and ethanol and oven-dried at 60 °C. The dried final material was crushed and sieved for further analysis. All the mechanisms involved are presented in ESI 1.†

### 2.5 Characterization

Fourier transform infrared (FT-IR) spectra were recorded using a PerkinElmer instrument between 4000 and 400 cm^−1^ with 32 scans in absorption mode. The samples were homogenized with KBr powder and pressed into pellets prior to analysis. The results revealed the functional groups present in the respective adsorbent material. The magnetic nature of the sample was analyzed at room temperature using a Lake Shore 7404 series vibrating sample magnetometer (VSM; McCorkle Boulevard, Westerville Ohio, USA). To analyze the crystallographic structure and the diffraction ability of the synthesized material, X-ray diffraction (XRD) analysis was performed on a Siemens D5000 X-ray diffractometer (Frimley, UK) using Cu Kα radiation (*λ* = 1.5418 Å) with a scan rate of 0.02 s^−1^ at 2*θ* = 10–90°; voltage: 40 kV; and current: 100 mA. Thermogravimetric analysis (TGA) was conducted to analyze the thermal stability of the synthesized material. This analysis was performed using a Q500 (PerkinElmer, Waltham, MA, USA). In this analysis, the sample was placed in an aluminum pan and heated over a temperature range of 25–900 °C at 10 °C min^−1^ under nitrogen purge. Transmission electron microscopy (TEM) analysis was performed to assess particle size, size distribution, and morphology using a Philips CM12 Version 3.3 TEM.

### 2.6 Adsorption study of 2,4-DNP and 2,4-DCP on Fe_3_O_4_@AC–SS adsorbent

The adsorption performance of Fe_3_O_4_@AC–SS was evaluated by investigating the effects of pH (3–10), contact time (5–120 min) and initial concentration of 2,4-DNP and 2,4-DCP (10–100 mg L^−1^). 20 mg of adsorbent (Fe_3_O_4_@AC–SS) was dispersed in 10 mL of 2,4-DNP and 2,4-DCP solution with the temperature of both analyte solutions kept at room temperature (298 K). The solutions were shaken at 250 rpm at constant temperature for the desired time. Next, the adsorbent was isolated from the solutions by applying a magnetic field. The residual concentration of 2,4-DNP and 2,4-DCP analytes in the supernatant was measured individually using UV-Vis spectrophotometry. Removal (%) and adsorption capacity (*q*_e_) of both analytes were calculated using eqn (1) and (2), respectively:1
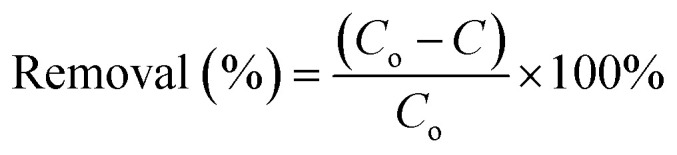
where *C*_o_ and *C* are the initial and final concentrations of 2,4-DNP and 2,4-DCP, respectively.2
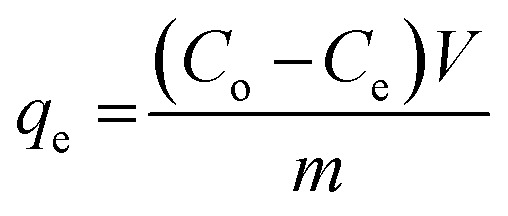
where *q*_e_ refers to the amount of analyte adsorbed (mg g^−1^), *C*_o_ and *C*_e_ are the initial and equilibrium concentrations of the liquid phase (mg L^−1^), respectively, *V* denotes the volume of solution (L), and *m* is the mass (g) of adsorbent used.

### 2.7 Preparation of environmental samples

The samples were collected from industrial areas located around Sungai Petani, Kedah, Malaysia (geographical coordinates: 5.6462° N, 100.5343° E) and Perai, Pulau Pinang, Malaysia (geographical coordinates: 5.3844° N, 100.3896° E). The collected water samples were filtered using nylon filters with a pore size of 0.45 μm.

## 3 Results and discussion

### 3.1 Characterization

#### 3.1.1 Fourier transform infrared spectroscopy

**Fig. 1:**
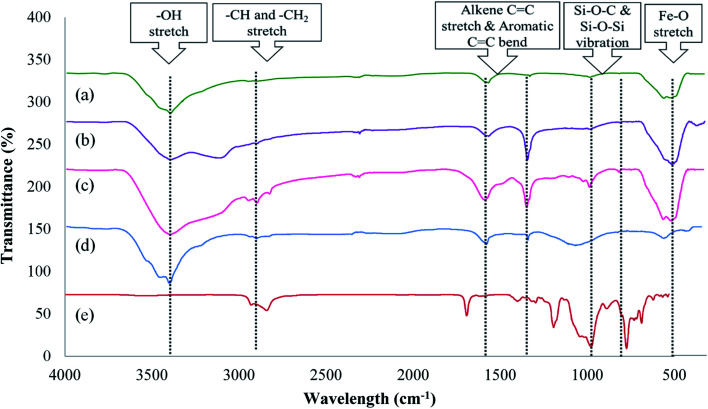
FTIR spectra of (a) Fe_3_O_4_ particles, (b) Fe_3_O_4_-AC, (c) Fe_3_O_4_@AC–SS, (d) AC, and (e) SS.

Before identifying the structural changes on the surface of AC, a preliminary investigation was conducted to determine the functional groups of SS as functionalizing agents and their magnetization characteristics. Hence, FTIR spectra were studied for all the materials and their individual components, as shown in Fig. 1(a)–(d). Pure Fe_3_O_4_ (Fig. 1(a)) exhibited strong bands at 3424 cm^−1^, 1619 cm^−1^, and 576 cm^−1^. The absorption band at 3424 cm^−1^ is attributed to the –OH stretching vibration, while the peaks recorded at 1619 cm^−1^ are due to the C=C stretch and H–OH bending vibration. The presence of an H–OH signal at this particular wavelength is due to the addition of water and ethanol during the synthetic process.^27^ The peak at 576 cm^−1^ is attributed to the Fe–O stretching vibration, which occurs due to the tetrahedral sites of the magnetic particles.^28^ The spectrum of Fe_3_O_4_-AC (Fig. 1(b)) exhibited peaks similar to that of Fe_3_O_4_, except for the additional peaks that appeared at 1617 cm^−1^ and 1500 cm^−1^ due to the presence of alkene –C=C stretch and –C=C aromatic stretching in the benzene rings, respectively, which are excessively present in AC. The formation of visible bands at these ranges proves that AC was successfully coated on the surface of the Fe_3_O_4_ particles.^29^ In the spectrum of the final material Fe_3_O_4_@AC–SS, as displayed in Fig. 1(c), the addition of SS changed the surface characteristics. Peaks were formed at 2981 cm^−1^, 2928 cm^−1^ and 2862 cm^−1^ due to –CH and –CH_2_ stretching, which are extensively present in the surfactant. Furthermore, the peaks formed at 880 cm^−1^, 634 cm^−1^, and 578 cm^−1^ are due to symmetric stretching and bending vibration of Si–O–Si from the surfactant used. The band near 1048 cm^−1^ is attributed to the Si–O–C stretching. The presence of these functional groups was compared to the individual spectrum of AC (see Fig. 1(d)) and SS (see Fig. 1(e)). The presence of silicone peaks in the spectrum prove that the silicone-based surfactant was coated successfully on the surface of the magnetic particles. The results are summarized and shown in ESI 2.†

#### 3.1.2 Vibrating sample magnetometry

**Fig. 2:**
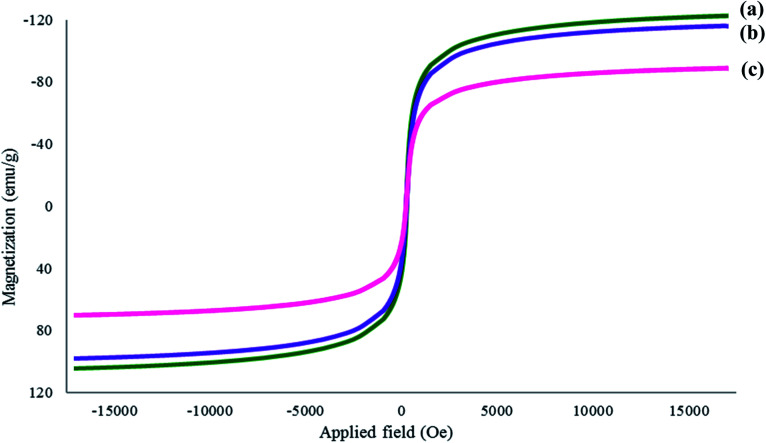
VSM magnetization curves of (a) Fe_3_O_4_ particles, (b) Fe_3_O_4_@AC and (c) Fe_3_O_4_@AC–SS.

VSM was employed in this study to examine the magnetic strength of the synthesized adsorbent. Fe_3_O_4_ particles, Fe_3_O_4_@AC and Fe_3_O_4_@AC–SS were examined to investigate their trend in magnetic strength upon coating. The hysteresis loops obtained are presented in Fig. 2(a)–(c) for the Fe_3_O_4_ particles, Fe_3_O_4_@AC and Fe_3_O_4_@AC–SS, respectively. Based on the results obtained, the magnetic saturation (*M*_s_) was observed at 115.28 emu g^−1^ for the Fe_3_O_4_ particles, 108.68 emu g^−1^ for Fe_3_O_4_@AC, and 80.76 emu g^−1^ Fe_3_O_4_@AC–SS. The magnetic strength appeared to deteriorate due to the presence of non-magnetic AC and silicone surfactant coating.^30^ This confirms that the coating process was successful. However, although the magnetization strength decreased upon coating the final material Fe_3_O_4_@AC–SS, its magnetic strength was still in the acceptable range and strong enough to perform in the adsorption procedure.^31^

#### 3.1.3 X-ray powder diffraction analysis

XRD analysis determines the characteristics and crystallinity of a material based on its diffraction ability. The XRD peaks for the Fe_3_O_4_ particles, Fe_3_O_4_@AC, Fe_3_O_4_@AC–SS and AC are shown in ESI 3.† The characteristic peaks at 2*θ* = 30.3°, 35.6°, 43.3°, 57.2°, and 62.9°, which corresponded to the (220), (311), (400), (511), and (440) cubic spinel planes of Fe_3_O_4_ based on JCPDS card number 65-3107, prove the presence of magnetic particles with high crystallinity in the material.^28^ The pattern of AC did not exhibit any distinctive peak due to the absence of magnetic particles on its surface. The peaks of Fe_3_O_4_@AC and Fe_3_O_4_@AC–SS displayed increased intensity and became sharper upon coating. This signifies the successful coating and increased crystalline phase upon the addition of AC and SS to the surface of the Fe_3_O_4_ magnetic particles.^32^

#### 3.1.4 Transmission electron microscopy analysis

The TEM images of synthesized materials and their respective cumulative particle size distribution histogram measured using the IMAGE J software are presented in ESI 4(a)–(d)† for Fe_3_O_4_, Fe_3_O_4_@AC, Fe_3_O_4_@AC–SS and AC, respectively. Nanoparticles are particles that exist with a size in the range of 1–100 nm. The porosity of particles is classified into three sizes namely microporous (<2 nm), mesoporous (2–50 nm) and macroporous (>50 nm). The mean particle sizes recorded for each adsorbent were Fe_3_O_4_ (22.9 nm), Fe_3_O_4_@AC (76.6 nm), Fe_3_O_4_@AC–SS (48.5 nm), and AC (81.8 nm). The particle sizes recorded for the Fe_3_O_4_ particles and Fe_3_O_4_@AC–SS were in the mesoporous region, while that of AC and Fe_3_O_4_@AC were in the macroporous region. The particle size of Fe_3_O_4_@AC had the tendency to increase due to the presence of AC, which is a macromolecule in nature (see ESI 4(b)†).^33^ Based on the results obtained all the adsorbents synthesized in this study were nanoparticles in size.

### 3.2 Effect of pH

**Fig. 3:**
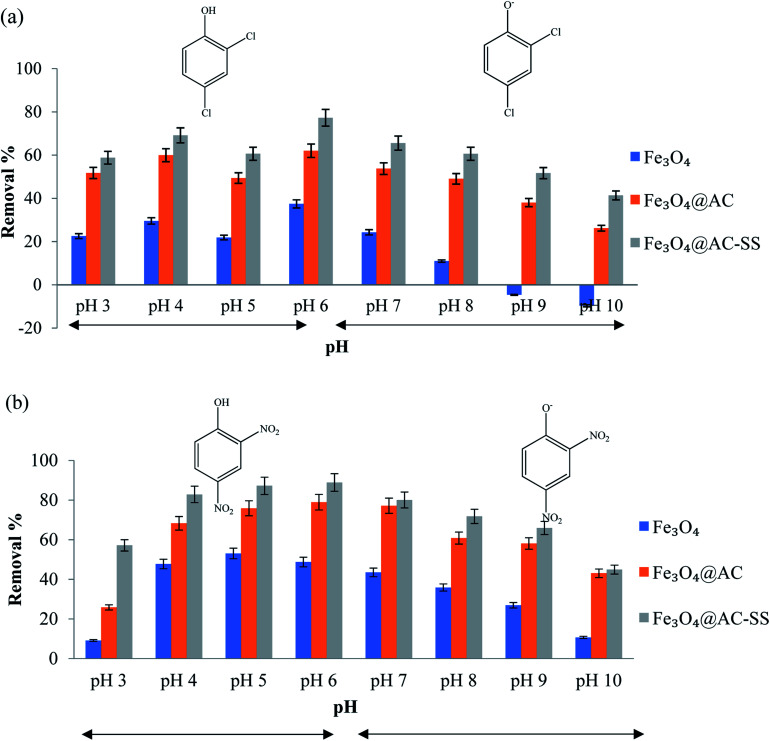
Effect of pH on (a) 2,4-DCP and (b) 2,4-DNP (conditions: adsorbent amount: 20 mg; analyte concentration: 10 mg L; and contact time: 60 min).

The pH of solution as one of the most important controlling parameters may affect the binding ability of the adsorbent during the adsorption process. Theoretically, pH can change the dissociation of functional groups on the active sites of the adsorbent, the surface charge of the adsorbent, and existing state of the adsorbate molecule.^34^ The p*K*_a_ values of the analytes were 2,4-DNP = 4.1 and 2,4-DCP = 7.9. Since the p*K*_a_ value of 2,4-DCP is higher, it is quite difficult to attain a lower pH. Hence, the optimization of both analytes was performed in the range of pH 3 to pH 10.^35^ The pH of the analyte solutions was adjusted using 0.01 M NaOH and 0.01 M HCl. This experiment was performed using the batch technique, in which each pH-adjusted analyte solution was shaken for 60 min in the presence of 20 mg of adsorbent separately. Fig. 3(a) and (b) display the removal trend for both analytes.

For 2,4-DCP, the removal percentage increased as the pH increased and started to decrease after the pH value exceeded 6. The analyte of 2,4-DCP is a weak acid and tends to exist in its protonated form when pH < p*K*_a_, while in the deprotonated form when pH > p*K*_a_.^36^ At higher pH medium, the increase in ionic and hydrophilic behavior of 2,4-DCP eventually decreases the solubilization, which causes a drastic drop in removal efficiency.^37^ The drastic decrease in removal percentage of 2,4-DCP when the pH was increased (pH 6 to pH 10) can be due to the anionic nature of the analyte, which is unable to form hydrogen bonds with the Fe_3_O_4_ particles.^35^ This eventually resulted in a drop in performance and negative removal percentage. The removal efficiency was better in the pH range of 5–7, particularly at its natural pH (pH = 6).

Similarly, 2,4-DNP displayed an increasing removal trend upon an increase in pH until pH 6, which decreased significantly upon a further increase in pH. Since 2,4-DNP is also a weak acid, it increased the uncharged 2,4-DNP concentration and decreased the charged 2,4-DNP concentration in the acidic medium. This causes 2,4-DNP to exist in the molecular form when the pH was reduced below its p*K*_a_ value.^38^ At pH > p*K*_a_ value, the 2,4-DNP molecule exists in its ionic form (phenolate ion). The repulsion that occurs between the phenolate ion and hydroxyl ion (–OH) on the adsorbent surface causes the removal to decline as the pH increases.^39^ pH 4, which is the natural pH of 2,4-DNP solution, was finalized as the optimum pH for this system since no significant increase was noted between the highest removal percentage (pH 6) and pH 4. The results of both analytes towards the adsorbent are depicted in Fig. 3.

In this study, the primary adsorption interactions were composed of hydrophobic interaction, hydrogen bonds and π–π interaction between the active binding sites and analyte. The π–π bond is formed between the electrons in the benzene rings of the phenolic compounds and AC surface, as illustrated in ESI 5.† The presence of electron withdrawing chloro- and nitro-groups in the phenolic compound increases the strength of the π–π interaction toward the material by decreasing the electron densities around the π electrons and reducing the repulsive electrostatic interaction between the aromatic rings.^40^ Since 2,4-DNP has high polar nature^41^ and stronger electron withdrawing capability^36^ compared to 2,4-DCP, the interaction of the nitro-phenolic compound is stronger than that of the chloro-phenolic compound.

### 3.3 Effect of contact time

**Fig. 4:**
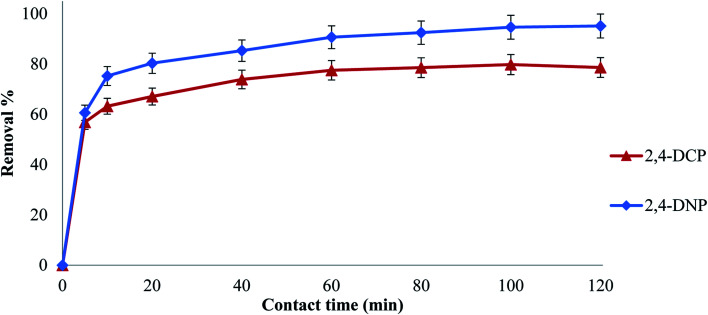
Effect of contact time on the removal of 2,4-DNP and 2,4-DCP (conditions: pH: 2,4-DCP = 6, 2,4-DNP = 4; adsorbent amount: 20 mg; and analyte concentration: 10 mg L^−1^) and kinetic best fitted pseudo-second order model graph.

Contact time is an essential optimization parameter, which provides the adsorption duration for maximal removal of a targeted analyte from an aqueous medium.^42^ Thus, to determine the equilibrium time for maximum uptake and to establish the kinetics of the adsorption process, contact time was investigated in the range of 5 to 120 min for both analytes. Fig. 4 shows that the maximum adsorption of 2,4-DCP and 2,4-DNP by Fe_3_O_4_@AC–SS was at approximately 60 min, and no further increase in adsorption was noted after 60 min.

For 2,4-DCP, the removal capacity of the final material (Fe_3_O_4_@AC–SS) increased drastically from 60–77% in the first hour and attained equilibrium after 60 min. For the controls, Fe_3_O_4_ particles and Fe_3_O_4_@AC, the removal increased initially and started to remain constant after 60 min with 37% and 64% removal, respectively. While for 2,4-DNP, the removal percentage of the final material (Fe_3_O_4_@AC–SS) increased from 60% to 90% in the first hour and reached a constant removal pattern with an increase in contact time. Meanwhile, for the control Fe_3_O_4_ particles and Fe_3_O_4_@AC the removal efficiency showed an increasing trend upon an increase in contact time until 60 min with the removal of 50% and 77%, respectively, and attained equilibrium with a further increase in contact time.

Based on the removal percentage performance, it can be concluded that the Fe_3_O_4_@AC–SS material displayed exceptional absorbency capacity compared to the unmodified Fe_3_O_4_ particles and Fe_3_O_4_@AC. The presence of OFX 0309 SS enhanced the adsorption capacity mainly due to the morphology and hydrophobic interaction toward both analytes. The Fe_3_O_4_@AC–SS material is highly porous, as obtained from SEM characterization.^26^ Based on the results obtained from SEM, it is highly visible that the coating increased the porosity and surface area of the Fe_3_O_4_@AC–SS material. Although the Fe_3_O_4_@AC–SS material is a nanomaterial and possesses a high surface area to volume ratio due to its porosity, the analytes were affected by the boundary layer attached to the binding site. This eventually increased the contact time.^43^

The removal efficiency of both analytes was drastic at the beginning of the removal due to the presence of many binding sites on the material surface. The presence of active binding sites enables the absorptive ions of the analytes to bind on the surface of the materials, thus enhancing the removal capacity.^44^ With an increase in contact time, the removal trend remained constant or was reduced for both analytes due to the saturation of analytes on the material surface. However, the unoccupied active sites became more difficult for the analytes to react^45^ due to the repulsion between the material surface and analyte. This caused the analytes to use extra energy to penetrate the surface of the material to gain access to the active sites, which eventually caused the removal percentage to decrease or become constant.^46^ Hence, 60 min was selected as the optimum contact time for both 2,4-DCP and 2,4-DNP.

### 3.4 Effect of adsorbent dosage

Optimization of adsorbent dosage is a key factor for adsorption equilibrium.^47^ In examining the effect of adsorbent dose on 2,4-DCP and 2,4-DNP removal, adsorption experiments were conducted with various amounts of Fe_3_O_4_@AC–SS adsorbent, ranging from 10–80 mg. ESI 6† shows the effect of adsorbent dosage on the removal efficiency.

A drastic increase was noted in the removal percentage when the adsorbent amount increased from 5 to 20 mg for both analytes. The availability of more binding sites and the absence of repulsion between the analytes increased the removal capacity proportionally with the increasing amount of adsorbent.^48^ The increase in adsorbent dosage after 20 mg had no drastic effect on the removal efficiency. Despite the vast active sites available with the increase in the amount of adsorbent, the conglomeration of adsorbent made it difficult for the analytes to react with the binding sites, which are overlapped. This resulted in no obvious variation in the removal percentage even after increasing the dosage amount.^49^ Hence, 20 mg was selected as the equilibrium dosage for both analytes.

### 3.5 Effect of initial concentration

The effect of initial concentration on the uptake of both analytes was determined by applying concentrations ranging between 10 and 100 mg L^−1^. The adsorbent behavior toward the increasing concentration for both 2,4-DCP and 2,4-DNP is exhibited in ESI 7.† The experiment was performed simultaneously for all concentrations. The adsorbent (20 mg) was mixed with analyte solution of varying concentrations and agitated using an orbital shaker for 60 min. The residual analyte solution was analyzed using a UV-vis spectrophotometer.

For 2,4-DCP, the removal efficiency increased rapidly up to 10 mg L^−1^ and decreased slightly at 20 mg L^−1^. Subsequently, the removal efficiency attained a constant value after 60 mg L^−1^. The saturation of analyte around the adsorbent results in competition for active sites, causing the removal percentage to become constant.^50^

Similarly, for 2,4-DNP, the removal efficiency increased rapidly up to 10 mg L^−1^ and declined in performance at 20 mg L^−1^. The removal efficiency slightly decreased after 60 mg L^−1^. The analytes tend to use energy to overcome the boundary layer effect, and thus a decrease in removal efficiency occurs after the saturation point.^43^ Therefore, the concentration of 60 mg L^−1^ was selected and was further used as the optimized concentration for both analytes.

### 3.6 Effect of solution temperature

In this removal study, the effect of temperature was studied to analyze the stability and binding capacity of the Fe_3_O_4_@AC–SS material with an increase in temperature. Starting from 298 K, the temperature was increased to 313 K, 333 K, 343 K, and 353 K for each analyte. The trend of binding capacity, *q*_e_ (mg g^−1^), decreased with an increase in temperature for both analytes based on the results obtained, and at a lower concentration (10–40 mg L^−1^), the variation in binding capacity was insignificant for all temperatures. At a higher concentration (60–100 mg L^−1^), the binding capacity was equilibrium at 298 K. This shows that the reaction involved was exothermic.^51^ The results indicate that an increase in temperature damages the active binding sites for the analytes and depletes the interaction between the analyte and adsorbent, which eventually decreases the removal efficiency.^38^ Hence, 298 K was selected as the optimal temperature in this study. All the respective graphs for this parameter are presented in ESI 8.†

### 3.7 Kinetic, isotherm and thermodynamic studies

#### 3.7.1 Adsorption kinetic models

The adsorption kinetic model was incorporated to describe the adsorption mechanism and the adsorption behavior of Fe_3_O_4_@AC–SS toward 2,4-DCP and 2,4-DNP. Studying the kinetics of adsorption helps determine the time required to reach equilibrium. This time is required in designing the batch adsorption systems. It offers valuable information about the mechanism of adsorption and the rate-determining step. The adsorption mechanism solely depends on the surface characteristic of the adsorbent used.^52^ Thus, to evaluate the kinetic data, some commonly used kinetic models were applied. The pseudo-first order^53^ and pseudo-second order^54^ models determine the rate of the adsorption process, while the Elovich model^55^ determines the reaction rate and the nature of the adsorption process. Intraparticle diffusion^56^ and the external model^57^ were employed to determine the rate-limiting step. Table 1 presents the results for 2,4-DCP and 2,4-DNP from the kinetic study.

**Table 1:** Details of the kinetic constants of various kinetic models for the adsorption process of 2,4-DCP and 2,4-DNP on Fe_3_O_4_@AC–SS

Kinetic model	Parameters	Material	
		Fe_3_O_4_@AC–SS	
		2,4-DCP	2,4-DNP
Pseudo-first-order	*q*_e_ (*q*_exp_) (mg g^−1^)	**4.0389**	**4.7505**
	*q*_e_ (*q*_cal_) (mg g^−1^)	0.9776	0.7773
	*K*_1_ (min^−1^)	0.0090	0.0122
	*R* ^2^	0.2258	0.2414
	Δ*q* (%)	32.9060	31.6120
	Relative error (%)	75.7950	83.6375
Pseudo-second-order	*q*_e_ (*q*_cal_) (mg g^−1^)	4.0700	4.8309
	*K*_2_ (g mg^−1^ min^−1^)	0.0597	0.0652
	*h* (mg g^−1^ min^−1^)	0.9880	1.5218
	*t* ^1/2^ (min)	0.0147	0.0297
	***R*** ^**2**^	**0.9969**	**0.9997**
	**Δ*q* (%)**	**0.2910**	**0.6397**
	**Relative error (%)**	**−0.7700**	**−1.6925**
Elovich equation	*q*_e_ (*q*_cal_) (mg g^−1^)	3.7256	4.5104
	*β* (g mg^−1^)	3.0807	2.1381
	*α* (mg g^−1^ min^−1^)	522.2000	120.2500
	*R* ^2^	0.9649	0.9528
	Δ*q* (%)	2.9318	1.9100
	Relative error (%)	7.7569	5.0500
Intra-particle diffusion	*C* (mg g^−1^)	2.7777	3.2038
	*K* (mg g^−1^ min^−1^)	0.1150	0.1569
	*R* ^2^	0.9481	0.8394
External diffusion	*K*_ext_ (min^−1^)	−0.0056	−0.0239
	*C* (mg g^−1^)	−0.9256	−1.3089
	*R* ^2^	0.9399	0.9361

The pseudo-first order model was initially introduced by Lagergren (1898) to study the kinetic behavior of liquid–solid adsorption. It helps to identify the adsorption rate by the adsorption capacity.^53^ This model states that the change in rate for analyte uptake is directly proportional to the variance in the saturation of concentration and amount of solid uptake.^58^ The respective equation for this model is described in eqn (3)–(5).3
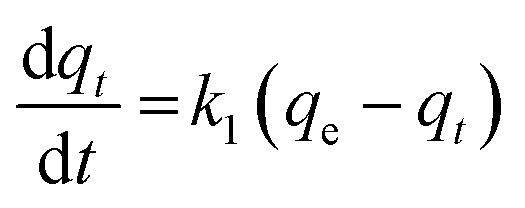

4 ln(*q*_e_ − *q*_t_) = log q_e_ − *k*_1_*t*

5
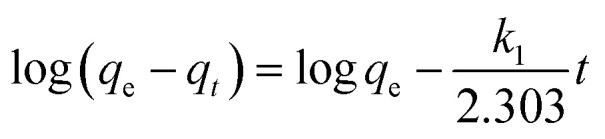
where *k*_1_ is first-order rate constant, *t* stands for contact time and *q*_e_ reflects the binding capacity. The values of log(*q*_e_ − *q*_*t*_) were calculated from the experimental data. The graph was plotted for log(*q*_e_ − *q*_*t*_) against *t* based on the calculations. Next, *k*_1_ was calculated from the slope of the plotted graph.

For the pseudo-first order model, it is clear that the calculated binding capacity (*q*_cal_) value was very far from the experimental data (*q*_exp_) (see Table 1). Thus, this study does not support this model. Additionally, the graph plotted for this model did not pass through the origin. This confirms that the data obtained in this study failed to fit this model and appears to be invalid for this research. In the case of the pseudo-second order, the kinetic study is performed to study the adsorption behaviors on solid phases.^54^Eqn (6) represents the pseudo-second order model:6
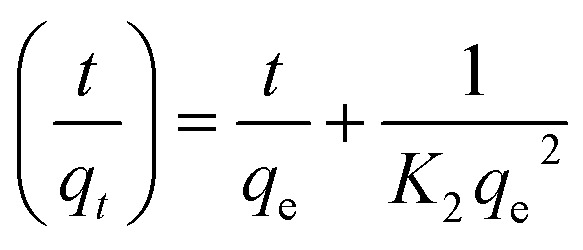
where *K*_2_ is the second-order rate constant, *t* represents contact time and *q*_e_ reflects the binding capacity. A graph was plotted for log *t*/*q*_*t*_ against *t*. Next, *K*_2_ was calculated from the slope of the plot.

**Fig. 5:**
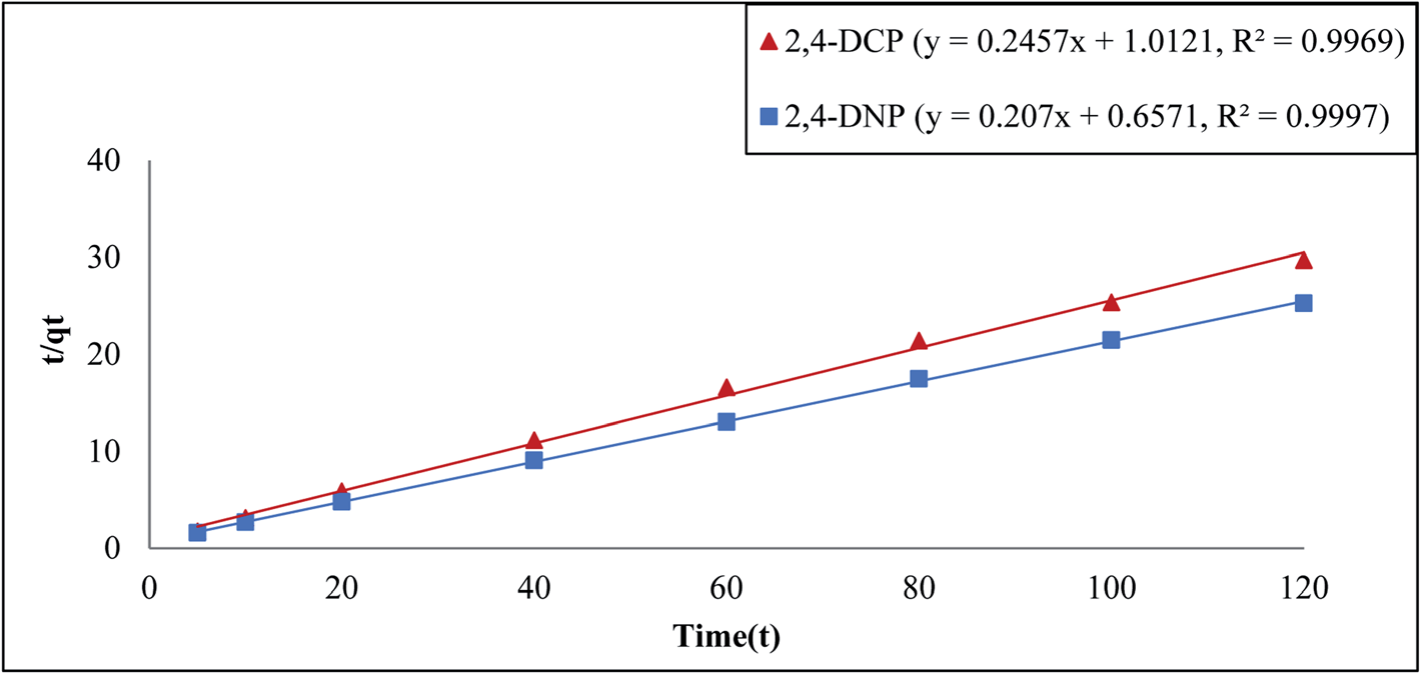
Adsorption pseudo-second-order model of Fe_3_O_4_@AC–SS towards 2,4-DCP and 2,4-DNP (conditions: pH: 2,4-DCP = 6, 2,4-DNP = 4; adsorbent amount: 20 mg; contact time: 60 min: analyte concentration: 60 mg L; and temperature 298 K).

Based on Table 1, the pseudo-second order model emerged as the most fitting model for both analytes in this study. The graph of this model is presented in Fig. 5. The values of Δ*q* and relative error (%) calculated for the pseudo-second order model were comparatively lower than that for the other models. The values of Δ*q* and relative error (%) calculated for the Fe_3_O_4_@AC–SS adsorbent are as follows: 2,4-DCP: (0.2910%, −0.7700%) and 2,4-DNP: (0.6397%, −1.6925%). The *R*^2^ values attained from the graphs plotted for the pseudo-second order model are 0.9969 (2,4-DCP) and 0.9997 (2,4-DNP), which support the adsorption capacity of the phenolic compounds toward the material. The calculated binding capacity (*q*_cal_) also seemed to be close with the experimental data (*q*_exp_), which confirms that the pseudo-second order is indeed the most suitable model for the Fe_3_O_4_@AC–SS adsorbent. When comparing both analytes, 2,4-DNP displayed better results and fit the pseudo-second order more than 2,4-DCP. This is attributed to the Fe_3_O_4_@AC–SS adsorbent, which is more favorable toward the nitro-group of the phenolic compound than the chloro-group due to its higher polarity^41^ and stronger electron withdrawing capability.^36^

Elovich models are basically used to study chemisorption kinetics.^55^ The Elovich equation is presented as eqn (7):7
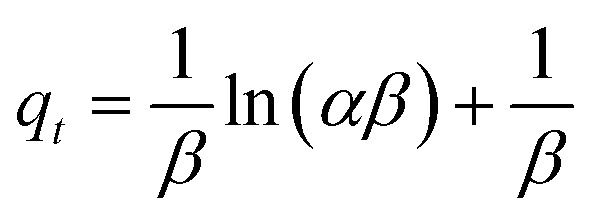
where *α* is the initial sorption rate, *β* represents the activation energy for chemisorption and *q*_*t*_ refers to the binding capacity. A graph was plotted for *q*_*t*_ against ln *t*.

Although the values of Δ*q* (%) and relative error (%) were not as low as the pseudo-second order model, the binding capacity of the Elovich model was the second best-fitted model with exceptional *R*^2^ values for 2,4-DCP (*R*^2^ = 0.9649) and 2,4-DNP (*R*^2^ = 0.9528). The results obtained for this model prove that a chemisorption process occurred between the adsorbent and analytes, in which this model fit the kinetic data for the adsorption of both target analytes.

Intraparticle diffusion is also known as the Weber and Morris model. This model normally deals with the adsorption behavior between bulk solutions and solid surfaces.^56^ The equation for this model is described in eqn (8):

8 *q*_*t*_ = *kt*^1/2^ + *c*

where *k* is the intraparticle diffusion rate constant, *t* refers to contact time and *c* represents the intercept. For this model, the graph was plotted for *q*_*t*_ against *t*^1/2^.

In this model, the intraparticle diffusion constant (*k*) is directly proportional to the boundary layer thickness.^59^ For this model, none of the straight lines plotted for this model intercept the origin. This proves that the intraparticle diffusion is not the only rate-limiting step, but other processes may be involved in the rate of adsorption.^35^ The larger the intercept of the graph, the higher the boundary layer effect (intercept: 2.7777 (2,4-DCP) and 3.2038 (2,4-DNP)).

Finally, the external diffusion model was applied to investigate the actual rate-controlling step involved in the adsorption process. The equation for this model is stated in eqn (9):9
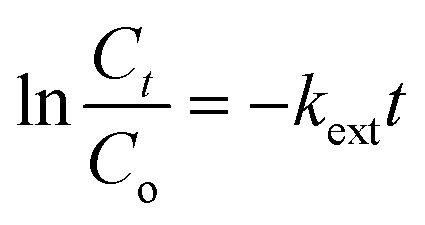
where *k*_ext_ refers to the external diffusion rate constant, and *t* represents contact time. A graph was plotted for ln *C*_*t*_/*C*_o_ against *t*. Similar to the intraparticle diffusion model, the graph plotted for this model does not intercept the origin. This supports the fact that external transport mainly governs the rate-limiting process of the analyte toward the Fe_3_O_4_@AC–SS adsorbent.^57^ The respective graphs for interparticle diffusion and external diffusion model are presented in ESI 9.†

In the study of kinetics, to justify the fitness of models, the normalized standard deviation Δ*q* (%) and relative error (%) must be calculated. The relative equations for both the normalized standard deviation and relative error are given below in eqn (10) and (11), respectively: 10
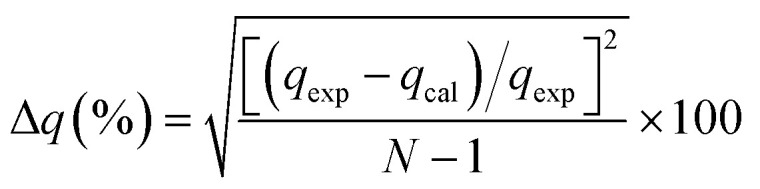
11
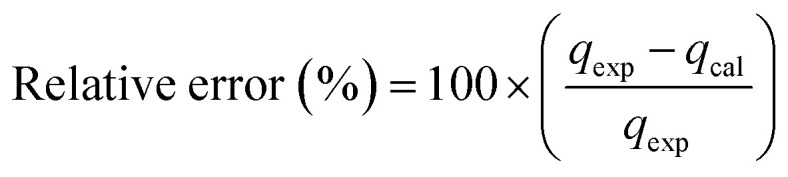
where *N* represents the number of data points used in the experiment, and *q*_exp_ (mg g^−1^) and *q*_cal_ (mg g^−1^) refer to the adsorption capacity from experiment and calculation, respectively.

#### 3.7.2 Adsorption isotherm model

The equilibrium data was fitted to several isotherm models to establish the most appropriate correlation for the adsorption system. These models were applied to fit the experimental equilibrium isotherm data of 2,4-DCP and 2,4-DNP adsorption on the Fe_3_O_4_@AC–SS adsorbent. In this removal study, five adsorption isotherm models were applied, including the Langmuir,^60^ Freundlich,^61^ Temkin,^62^ Halsey^63^ and Dubinin–Radushkevich^64^ models. For the isotherm model analysis, the Fe_3_O_4_@AC–SS adsorbent behavior was analyzed at five temperatures, 298 K, 313 K, 333 K, 343 K, and 353 K. The results calculated based on the five temperatures for both analytes are presented in Table 2.

**Table 2:** Details of the isotherm constants determined for various adsorption isotherm models for the adsorption process of 2,4-DCP and 2,4-DNP on the adsorbent Fe_3_O_4_@AC–SS

Analyte	Parameters	Temperature				
		298 K	313 K	333 K	343 K	353 K
**Langmuir**						
2,4-DCP	*q*_m_ (mg g^−1^)	98.0392	84.8176	89.2857	73.5294	52.083
	*b* (L mg^−1^)	0.0123	0.0196	0.0113	0.0122	0.0154
	*R* ^2^	0.9504	0.9017	0.7081	0.8770	0.9085
	*R*_L_	0.5066	0.4591	0.5953	0.5774	0.5204
2,4-DNP	*q*_m_ (mg g^−1^)	46.0829	49.7512	43.4783	26.9542	14.8588
	*b* (L mg^−1^)	0.0894	0.0435	0.0440	0.4294	0.0360
	*R* ^2^	0.7851	0.9089	0.7727	0.9302	0.9595
	*R*_L_	0.1572	0.2772	0.2749	0.2796	0.3166

**Freundlich**						
2,4-DCP	*K*_F_ ((mg g^−1^) (L mg^−1^)^1/*n*^)	1.8425	2.0086	1.2075	1.0607	0.9513
	***N***	**1.1942**	**1.3002**	**1.1435**	**1.1368**	**1.1482**
	1/*n*	0.8374	0.7691	0.8745	0.8797	0.8709
	***R*** ^**2**^	**0.9998**	**0.9988**	**0.9976**	**0.9995**	**0.9994**
2,4-DNP	*K*_F_ ((mg g^−1^) (L mg^−1^)^1/*n*^)	3.9201	2.3823	2.1188	1.4876	0.7125
	***N***	**1.6234**	**1.3583**	**1.3405**	**1.3454**	**1.2404**
	1/*n*	0.6160	0.7362	0.7460	0.7433	0.8062
	***R*** ^**2**^	**0.9719**	**0.9929**	**0.9815**	**0.9971**	**0.9977**

**Temkin**						
2,4-DCP	*K*_T_ (L mg^−1^)	0.4153	0.4750	0.3097	0.3050	0.2770
	*b*_T_ (kJ mol^−1^)	210.3738	265.7966	261.7283	244.3827	316.8486
	*R* ^2^	0.9227	0.9178	0.9140	0.9338	0.9294
2,4-DNP	*K*_T_ (L mg^−1^)	0.8824	0.5176	0.4667	0.3348	0.2011
	*b*_T_ (kJ mol^−1^)	269.7617	253.6584	274.6317	320.2576	406.0323
	*R* ^2^	0.9078	0.9417	0.9478	0.9195	0.9295

**Dubinin–Radushkevich (D–R)**						
2,4-DCP	*q*_m_ (mg g^−1^)	22.7031	20.1600	19.6465	18.6156	17.1124
	*β* (mol^2^ kJ^−2^)	2.7610	1.9817	3.5910	4.3731	4.2337
	*R* ^2^	0.7825	0.7666	0.7766	0.7747	0.7700
	*E*	1.2036	1.4207	1.0554	0.9564	0.9720
2,4-DNP	*q*_m_ (mg g^−1^)	21.8106	21.5118	20.6559	17.6547	13.6031
	*β* (mol^2^ kJ^−2^)	0.7336	1.6375	1.7332	2.8632	8.2548
	*R* ^2^	0.6595	0.7383	0.7288	0.7207	0.7877
	*E*	2.3351	1.5629	1.5192	1.1820	0.6961

**Halsey**						
2,4-DCP	*N*	−1.1942	−1.3000	−1.1435	−1.1368	−1.1423
	*K*	0.4821	0.4038	0.8060	0.9352	1.0591
	*R* ^2^	0.9998	0.9988	0.9976	0.9995	0.9994
2,4-DNP	*N*	−1.6234	−1.3583	−1.3405	−0.1345	−1.2404
	*K*	0.4311	0.5278	0.5712	0.7443	0.2732
	*R* ^2^	0.9719	0.9929	0.9815	0.9971	0.9977

The Langmuir isotherm model is a frequently applied model in most isotherm studies. It describes the energy that has been adsorbed onto the surface and the movements of adsorbate on the surface of a material. This theory basically describes adsorption that occurs on a homogeneous surface.^58^ To further analyze the suitability of this model, the *R*_L_ dimensional separation factor was calculated.^1^ The equation for this model and the equation for the dimensional separation factor are described in eqn (12) and (13), respectively:12
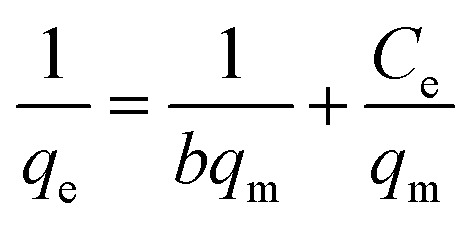
13
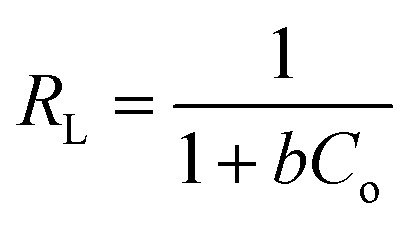
where *q*_e_ represents the adsorption capacity (mg g^−1^), *C*_e_ is the equilibrium concentration of adsorbate, *C*_o_ refers to the initial concentration of adsorbate, and *q*_m_ and *b* are the adsorption capacity and rate of adsorption, respectively. The graph for this model was plotted for *C*_e_/*q*_e_ against *C*_e_. The basic concept of the Langmuir model states that when the formation of a monolayer occurs on the surface of an adsorbent, only one analyte can be adsorbed in the active sites, and the intermolecular forces decrease with distance.^62^ Based on Table 2, the adsorption capacity (*q*_e_) calculated was not in agreement with the experimental values. The coefficient of determination (*R*^2^) recorded was relatively low compared to the other models studied, confirming that this model does not offer a satisfactory fit for this study.

The Freundlich model is also a frequently applied model in isotherm studies. This model investigates the adsorption capacity of heterogeneous surfaces.^62^ The equation for this model is stated in eqn (14):14
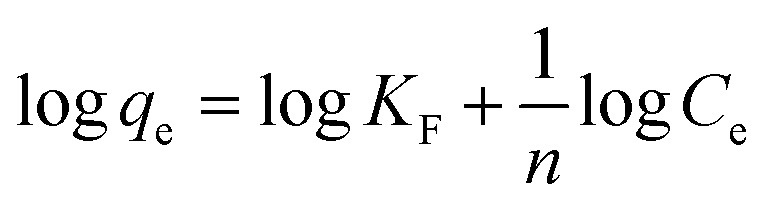
where *q*_e_ represents the adsorption capacity (mg g^−1^), *C*_e_ is the equilibrium concentration, and *K*_F_ and *n* are isotherm constants for capacity and intensity, respectively. The graph for this model was plotted for log *q*_e_ against log *C*_e_.

**Fig. 6:**
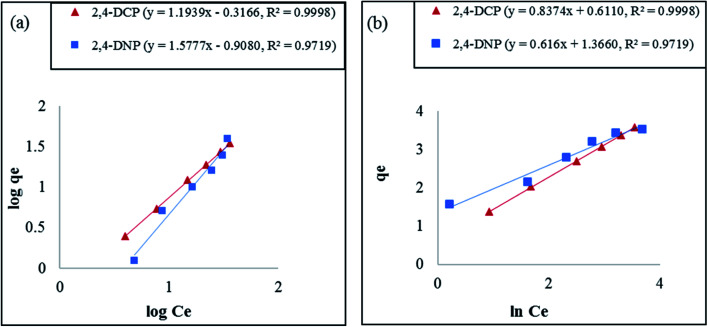
Adsorption isotherms of (a) Freundlich and (b) Halsey models for Fe_3_O_4_@AC–SS towards 2,4-DCP and 2,4-DNP at 298 K (conditions: pH: 2,4-DCP = 6, 2,4-DNP = 4; adsorbent amount: 20 mg; contact time: 60 min; and analyte concentration: 60 mg L^−1^).

Based on the results tabulated in Table 2, the isotherm model that best fits this removal study is the Freundlich model. The *R*^2^ value recorded did not exceed 0.9719 for both analytes at all temperatures. The Freundlich model is normally applied for multilayer adsorption, non-uniform heat distribution, and affinities over heterogeneous surfaces.^61^ The decrease in Freundlich equilibrium constant for capacity (*K*_F_) with an increase in temperature proves the reaction was exothermic.^1^ The equilibrium constant for intensity (1/*n*) recorded for this model, which ranged from 0 to 1, signifies that the phenolic compounds are constructively adsorbed onto the surface of Fe_3_O_4_@AC–SS.^65^ The plot for this model at 298 K is shown in Fig. 6(a).

In the case of the Temkin isotherm model, its explains that the adsorption temperature of materials decreases with the interaction between the adsorbent and adsorbate.^62^ The equations for this model are displayed in eqn (15) and (16):

15 *q*_e_ = *β* ln *K*_T_ + *β* ln *C*_e_

16
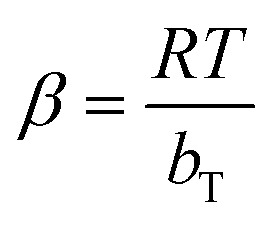
where *q*_e_ represents the adsorption capacity (mg g^−1^), *C*_e_ is the equilibrium concentration, *K*_T_ refers to the isotherm intercept and *b*_T_ denotes the heat of adsorption. The graph for this model was plotted for *q*_e_ against ln *C*_e_. For this model, the heat of adsorption of all the molecules on the adsorbent surface decreased linearly with coverage.^66^ This proves that the Fe_3_O_4_@AC–SS adsorbent is made up of multiple layers. In this model, the *R*^2^ values recorded for both analytes exceeded 0.9078 for all the studied temperatures. The heat of sorption, *b*_T_ (kJ mol^−1^), seemed to increase from 210.3738 to 316.8486 for 2,4-DCP and from 269.6170 to 406.0323 for 2,4-DNP, upon an increase in temperature.

The next isotherm model refers to the Halsey model. This model mainly describes the heteroporous nature of a material.^63^ The equation for this formula is explained in eqn (17):17
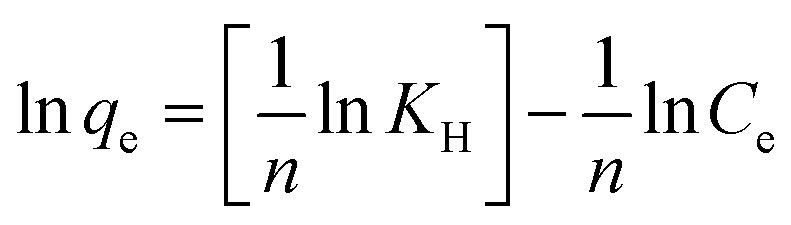
where *q*_e_ denotes the adsorption capacity (mg g^−1^), *C*_e_ stands for the equilibrium concentration, and *K*_H_ is the Halsey isotherm constant. The graph for the Halsey model was plotted for ln *q*_e_ against ln *C*_e_. The *R*^2^ recorded for the Halsey model exceeded 0.9719, displaying the heteroporous structure of the Fe_3_O_4_@AC–SS adsorbent. The respective graph for this model at 298 K is shown in Fig. 6(b).

The other model tested in this study was the Dubinin–Radushkevich model. This model describes the adsorption of gasses and vapors on a microporous solid.^66^ This model is related to the porous structure of the adsorbent used.^66^ The equations for this formula are given in eqn (18) and (19):

18 ln *q*_e_ = ln *q*_m_ − *βE*^2^

19 *E* = *RT* ln[1 + 1/*C*_e_]

where *q*_e_ represents the adsorption capacity (mg g^−1^), and *β* denotes the adsorption energy constant. The graph for the Dubinin–Radushkevich model was plotted for ln *q*_e_ against ln *E*^2^. Based on the data tabulated in Table 2, the coefficient of determination, *R*^2^, was relatively low when compared to the other models, thus proving that this model poorly fits the experimental data in comparison to the other studied models.

Since both the analytes fit the Freundlich model, this verified that the material used in this study has a heterogeneous surface with multiple functional groups (–OH, –COOH, –Si–O, and aromatic rings) on its surface.^62^ Thus, it can be concluded that there are interactions, such as hydrogen bonding, hydrophobic interaction and strong π–π interactions, involved between the adsorbent and adsorbate.^36^ Based on the *R*^2^ values, the models that best fit this adsorption study are presented in the following order: Freundlich, Halsey > Temkin > Langmuir > Dubinin–Radushkevich for 2,4-DCP and 2,4-DNP.

#### 3.7.3 Thermodynamic study

The thermodynamic study was conducted to analyze the behavior of the material towards the analytes at varying temperatures. In this thermodynamic study, five temperatures were set, starting from room temperature 298 K, to 313 K, 333 K, 343 K, and 353 K. The results for enthalpy Δ*H*°, entropy Δ*S*°, and Gibbs free energy Δ*G*° are shown in ESI 10† for 2,4-DCP and 2,4-DNP. The thermodynamic parameters Δ*G*°, Δ*H*°, and Δ*S*° were determined using eqn (20)–(22):^67^

20 Δ*G*° = −*RT* ln *K*_d_

21
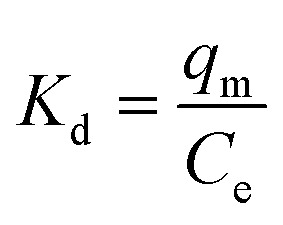
22
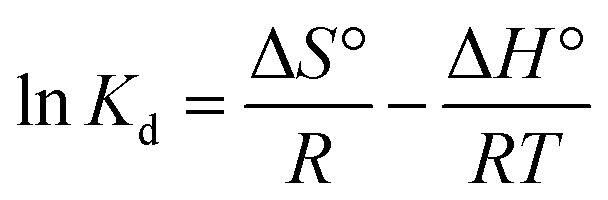
where, *K*_d_ is the equilibrium constant, *q*_m_ denotes the amount of analyte (mg) adsorbed on the adsorbent at equilibrium, *C*_e_ (mg L^−1^) is the equilibrium concentration, *T* (K) represents solution temperature and *R* is the gas constant. The values of Δ*H*° and Δ*S*° were obtained from the slope and intercept of the plot of ln *K*_d_ against 1/*T*, respectively.

The value of Δ*G*° was negative at 298 K and 313 K for the 2,4-DCP analyte and started turning positive after the temperature increased to 333–353 K. This shows that the reaction was spontaneous at lower temperature and thermodynamically favorable.^4^ With a further increase in temperature from 333–353 K, the Gibbs free energy Δ*G*° turned positive, indicating a non-spontaneous chemical reaction. In ESI 10†, the Δ*G*° recorded for 2,4-DNP showed a negative value for temperatures in the range of 298–333 K. An increase in temperature from 343–353 K resulted in a positive value for Δ*G*°. This proves that the reaction for 2,4-DNP is also spontaneous at lower temperature, while unfavorable at high temperature. The summarized thermodynamic results for both 2,4-DCP and 2,4-DNP are shown in ESI 10.† The thermodynamic Van't Hoff plot is shown in ESI 11† for both analytes.

For Δ*H*°, the values recorded for both the analytes were negative, where for 2,4-DCP and 2,4-DNP they were −9.7091 and −20.1615, respectively. The negative value of Δ*H*° showed that the reaction was exothermic.^4^ This is supported by the Freundlich model isotherm results retrieved from the isotherm study, which prove that the reaction was indeed an exothermic reaction with an increase in temperature.^1^

The values for Δ*S*° were −30.9630 and −62.8829 for 2,4-DCP and 2,4-DNP, respectively. The negative values of Δ*S*° show the decreased randomness between the adsorbate and adsorbent during the sorption process. This also verifies that no significant change occurred in the internal structure of the adsorbent when the temperature increased.^1^ Compared to the Δ*S*° value for both analytes, the value of 2,4-DNP was higher than that for 2,4-DCP. This proves that the Fe_3_O_4_@AC–SS material was more favorable towards 2,4-DNP.

### 3.8 Reusability study

The reusability of adsorbents is an important process for economical and resource reasons. To be useful in the removal process, the adsorbed species should be easily desorbed under suitable conditions and the adsorbents should be used many times in order to reduce material costs. Thus, adsorbent reusability and the desorption of 2,4-DCP and 2,4-DNP loaded on Fe_3_O_4_@AC–SS were assessed using five adsorption–desorption cycles. Acetone solution was used as the desorption agent to remove the adsorbed 2,4-DCP and 2,4-DNP molecules. After the desorption process, the material was separated using an external magnetic field and was dried completely prior to the next cycle. ESI 12† presents the percentage removal after six cycles for both 2,4-DCP and 2,4-DNP. The removal% was almost constant for the first 3 cycles with a removal efficiency of 78% (2,4-DCP) and 89% (2,4-DNP). For the fourth cycle, the removal efficiency decreased slightly due to leaching of the material surface properties during the desorption process, hence deteriorating the removal performance.^68^ The reusability studies of the material showed that the material can be reused for up to three cycles with a removal efficiency of 82.9% (2,4-DNP) and 74.4% (2,4-DCP). This proves that the material can serve as an economical and effective adsorbent for the removal of chloro- and nitro-group phenolic compounds from aqueous solutions in industrial applications.

### 3.9 Comparison of adsorption capacities

The Fe_3_O_4_@AC–SS material was compared with other materials used by other researchers in past studies. The results are summarized in Table 3. The material used in this study has a relatively higher sorption capacity (*q*_e_) with lower equilibrium time compared to the other research studies. This strengthens the status of the material to be applied as a sorbent for phenolic compounds.

**Table 3:** Comparison of the binding capacity and contact time of the Fe_3_O_4_@AC–SS material with other materials from other research studies

Adsorbent	Adsorbate	Sorbent dosage (g)	*C*_o_ (mg L^−1^)	Maximum capacity *q*_m_ (mg g^−1^)	*q*_*t*_ (hours)	Reference
Coconut coir pith	2,4-DCP	0.1	50	19.12	0.3	65
Palm pith carbon	2,4-DCP	0.05	10	19.16	1	4
Pomegranate peel	2,4-DCP	10	106	75.80	9	16
Bamboo based activated carbon	2,4-DNP	—	23.4	0.891	24	18
Activated carbon fibers	2,4-DNP	0.1	270	1.49	24	36
**Fe**_**3**_ **O**_**4**_ **@AC–SS**	**2,4-DNP**	0.02	60	**46.08**	1	**This work**
	**2,4-DCP**	0.02	60	**98.04**	1	

### 3.10 Precision and reproducibility

To further validate the proposed method, linearity and reproducibility experiments were carried out for both analytes. The linearity curve was plotted by spiking the analyte solution in industrial wastewater with a concentration ranging between 1 and 60 mg L^−1^. Inter-day precision and accuracy experiments were performed by spiking three levels of concentrations (1, 10 and 40 mg L^−1^) to the industrial wastewater sample. The analysis was performed seven times (*n* = 7) and on triplicate sample. The mean values of removal percentage for 2,4-DCP and 2,4-DNP were 88.33 and 97.22 for 1 mg L^−1^, 79.13 and 84.98 for 10 mg L^−1^, and 68.13 and 78.12 for 40 mg L^−1^, respectively. The precision results for all three concentrations were recorded as a percentage of the relative standard deviation value (RSD%). The RSD% values for inter-day and intra-day for 2,4-DCP are in the range of 0.65–5.84% and 1.65–5.91%, respectively. For 2,4-DNP, the RSD% for inter-day and intra-day was recorded in the range of 2.88–3.91 and 2.46–5.81, respectively. The validation data are summarized in ESI 13.†

### 3.11 Analysis of real samples

Real sample analysis was conducted to investigate the effect of sample matrices on the removal efficiency of the Fe_3_O_4_@AC–SS adsorbent toward phenolic compounds. The sample analysis was conducted under optimized conditions in triplicate. Each sample was spiked with analyte solution at concentrations of 1, 10 and 40 mg L^−1^. The results obtained are recorded in Table 4 for both analytes with the respective removal percentage and RSD%. The mean removal percentage values recorded for 2,4-DCP ranged from 62.23% to 87.47% with RSD% ranging from 0.0%2 to 7.20%. For 2,4-DNP, the mean recovery ranged from 70.49% to 98.09% with RSD% below 6.52%.

**Table 4:** Removal efficiency of 2,4-DCP and 2,4-DNP in various real samples towards the Fe_3_O_4_@AC–SS adsorbent

Industrial wastewater	Spiked levels (mg L^−1^)	2,4-DNP				2,4-DCP			
		Found (mg L^−1^)	Adsorbed (mg L^−1^)	Removal (%)	RSD (%)	Found (mg L^−1^)	Adsorbed (mg L^−1^)	Removal (%)	RSD (%)
Plastic industry	1	1.51	1.30	85.79	0.44	0.85	0.68	79.86	4.09
	10	11.53	9.56	82.93	0.07	10.70	7.52	70.32	3.01
	40	42.64	30.60	71.77	5.14	40.61	25.62	63.09	1.85
Glove industry	1	1.83	1.64	89.97	3.50	0.93	0.75	81.28	0.78
	10	11.14	9.70	87.10	0.00	11.75	9.03	76.83	0.02
	40	40.57	32.51	80.13	1.25	41.04	27.61	67.26	1.00
Wood industry	1	1.36	1.26	92.71	0.48	1.12	0.98	87.47	1.69
	10	11.27	9.62	85.44	0.46	12.41	9.47	76.34	0.26
	40	39.60	31.16	78.70	0.31	40.19	28.12	69.95	1.64
Pesticide industry A	1	0.89	0.73	82.46	0.26	0.91	0.71	78.24	3.56
	10	11.32	8.78	77.55	0.12	10.50	7.08	67.46	2.63
	40	42.51	29.97	70.49	0.32	40.44	25.17	62.23	7.20
Pesticide industry B	1	1.10	1.08	98.09	6.52	1.00	0.86	86.23	1.30
	10	11.09	9.89	89.16	0.01	9.48	7.39	77.84	0.05
	40	42.36	33.71	79.58	0.28	41.72	28.24	67.69	4.03

## 4 Conclusion

To the best of our knowledge, this is the first report on the use of a non-ionic silicone surfactant (OFX0309) as a material to modify the Fe_3_O_4_@AC surface for the removal of 2,4-DCP and 2,4-DNP. Non-ionic silicone surfactant (OFX0309) was successfully used for developing a new type of magnetic activated carbon capable of removing chloro- and nitro-group phenolic compounds effectively. The effect of silicone surfactant was investigated in the removal of 2,4-DCP and 2,4-DNP from aqueous samples. 2,4-DCP and 2,4-DNP were adsorbed in batch mode, and the effect of various operating conditions, such as pH, contact time, adsorbent dosage, initial concentration of both analytes and temperature, were investigated. The experimental results show that high removal percentage was obtained at the optimized parameters: pH 6 (2,4-DCP) and pH 4 (2,4-DNP); 60 min as the optimum contact time; 20 mg of adsorbent dosage; 60 mg L^−1^ for the optimized initial concentration; and 298 K as the optimized temperature. Fe_3_O_4_@AC–SS exhibited better removal efficiency compared to Fe_3_O_4_@AC. The presence of silicone surfactant revealed that its enhanced adsorptive capacity was due to hydrophobic interaction, hydrogen bonds and π–π interaction between the binding sites and analytes. However, 2,4-DNP showed higher adsorption capacity due to its stronger electron withdrawing capability than 2,4-DCP.

The thermodynamic parameters Δ*G*°, Δ*S*° and Δ*H*° for the adsorption of both analytes on Fe_3_O_4_@AC–SS were determined. Based on the results obtained in the thermodynamic study, the negative Δ*H*° showed that the reaction involved between adsorbent and analyte was exothermic. The negative value of Δ*S*° portrayed a decrease in randomness between the adsorbent and analyte during the sorption process. The conversion of negative Δ*G*° value to positive value upon an increase in temperature showed that the material is thermodynamically stable, and the reaction favors room temperature for both analytes. The Freundlich isotherm was found to be better for the adsorption of both analytes on Fe_3_O_4_@AC–SS. The pseudo-second order kinetic model successfully explains the kinetic data.

To further validate the optimized method, it was applied to real samples to study the linearity and reproducibility. The matrix effect was analyzed by applying the optimized system on real industrial wastewater samples. Both 2,4-DCP and 2,4-DNP obtained mean removal% values in the range of 62.23–87.47% and 70.49–98.09%, respectively. Also, compared to other studies based on activated carbon coated with surfactant, this type of silicone surfactant was unequivocally shown to be non-toxic to normal and cancer cells, as tested by our group (unpublished data). Therefore, the non-ionic silicone surfactant combined with the Fe_3_O_4_@AC material has great potential to be explored for removing organic pollutants from water samples based on their toxicity and unique structure molecules that could entrap hydrophobic and hydrophilic substances. Considering the great properties of this hybrid material, it can be assured that Fe_3_O_4_@AC–SS can be used an excellent adsorbent in the removal of other existing harmful organic pollutants.

## Conflicts of interest

There are no conflicts to declare.

## Supplementary Material

RA-009-C9RA07151B-s001
