# Supplementary material for: Development of a new efficient and economical magnetic sorbent silicone surfactant-based activated carbon for the removal of chloro- and nitro-group phenolic compounds from contaminated water samples
Source: RSC Adv. 2019 Nov 13;9(63):36915–30. doi: 10.1039/c9ra07151b (PMC9075134; doi:10.1039/c9ra07151b)
Supplement: RA-009-C9RA07151B-s001 [file RA-009-C9RA07151B-s001.pdf]

Electronic Supplementary Material (ESI) for RSC Advances.
This journal is © The Royal Society of Chemistry 2019

Supplementary data

SD 1: Schematic preparation of a) Magnetic Fe_3O_4 particles, b) AC and SS complex, and c) Fe_3O_4@AC-SS.

SD 2: Summarized FTIR readings.

SD 3: XRD spectra of (a) Fe_3O_4 particles, (b) Fe_3O_4-AC, (c) Fe_3O_4@AC-SS and (d) AC.

SD 4: TEM images of (a) Fe_3O_4 particles, (b) Fe_3O_4-AC, (c) Fe_3O_4@AC-SS, (d) AC and their respective cumulative particle distribution graphs.

SD 5: Possible proposed adsorption mechanism for 2,4-DCP and 2,4-DNP towards Fe_3O_4@AC-SS.

SD 6: Effect of adsorbent dosage on the removal of 2,4-DCP and 2,4-DNP (Condition: pH: 2,4-DCP = 6, 2,4-DNP = 4; contact time: 60 minutes; analyte concentration: 10 mg/L).

SD 7: Effect of initial concentration on the removal of (a) 2,4-DCP and (b) 2,4-DNP (Condition: pH: 2,4-DCP = 6; 2,4-DNP =4; contact time: 60 minutes; adsorbent dosage: 20 mg).

SD 8: Effect of temperature on the removal of (a) 2,4-DCP and (b) 2,4-DNP (Condition: pH: 2,4-DCP = 6; 2,4-DNP = 4; contact time: 60 minutes; adsorbent dosage: 20 mg).

SD 9: a) Kinetic intraparticle diffusion model and b) external diffusion model of 2,4-DCP and 2,4-DNP.

SD 10: Details of thermodynamic parameters for the adsorption of 2,4-DCP on Fe_3O_4@AC-SS.

SD 11: Thermodynamic Van Hoff plot for the adsorption of 2,4-DCP and 2,4-DNP on Fe_3O_4@AC-SS.

SD 12: Reusability cycles for 2,4-DCP and 2,4-DNP.

SD 13: Validation data on the removal study of 2,4-DCP and 2,4-DNP.

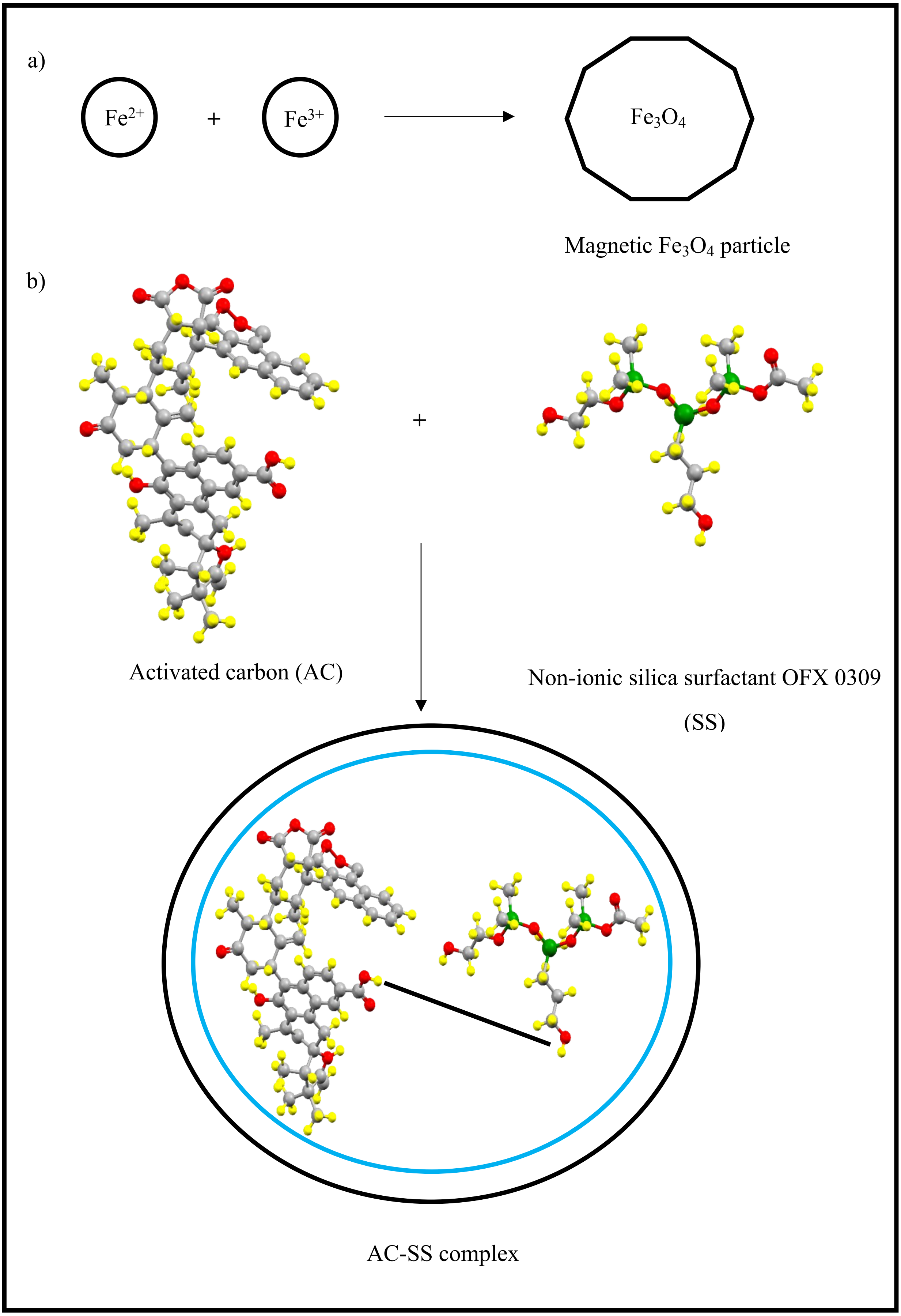

a)
Fe2+
+
Fe3+
Fe3O4
Magnetic Fe3O4 particle
b)
+
Activated carbon (AC)
Non-ionic silica surfactant OFX 0309
(SS)
AC-SS complex

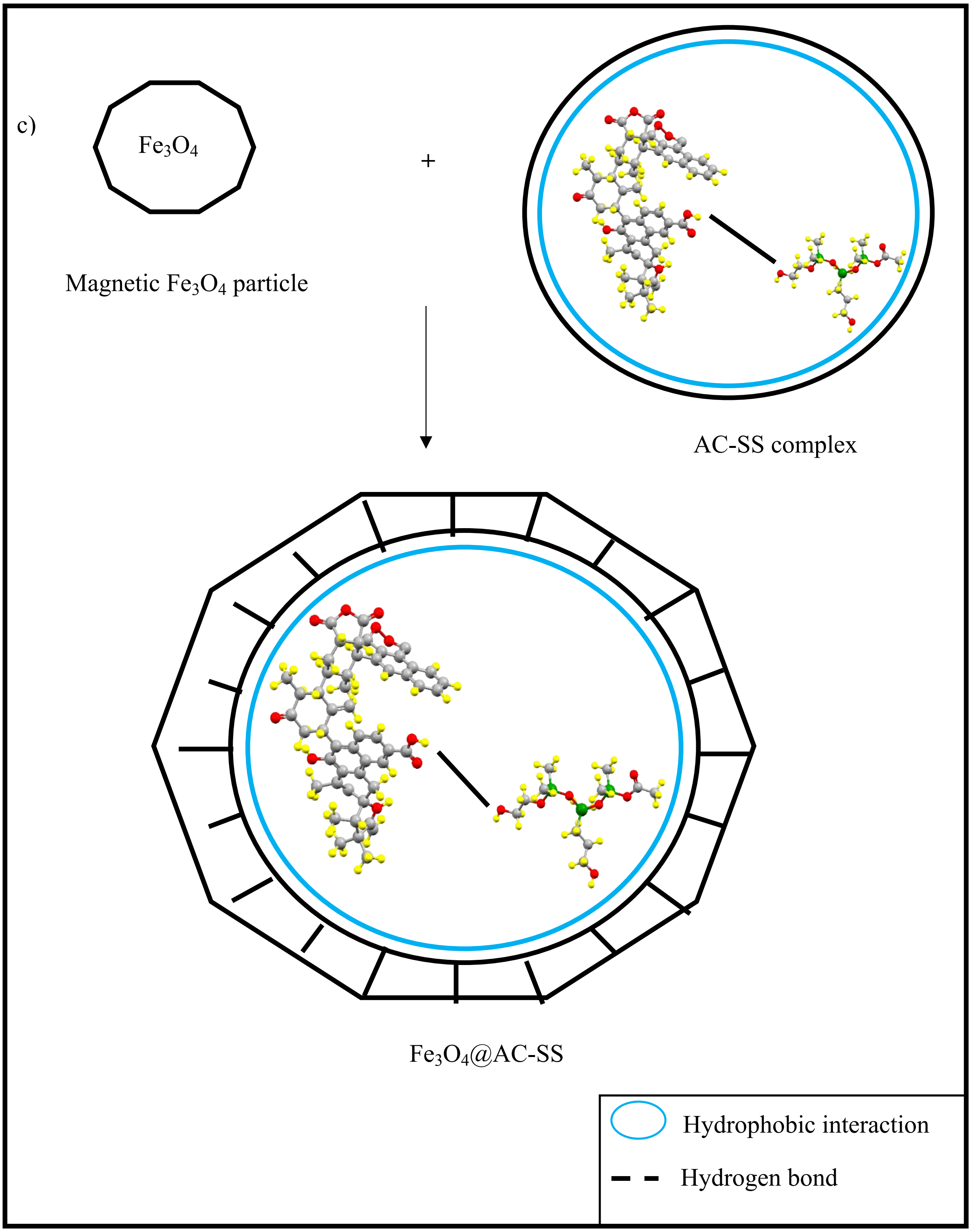

SD 1: Schematic preparation of a) Magnetic Fe_3O_4 particles, b) AC and SS complex, and c) Fe_3O_4@AC-SS.

SD 2: Summarized FTIR readings.

Characterization	Fe_3O_4	Fe_3O_4-AC	Fe_3O_4@AC-SS	AC	SS
**a) FTIR (cm^{-1})**					
• OH stretching	3416	3418	3415	3419	-
• Alkene C=C stretch & H-OH bending	1615	1619	1619	1618	-
• Aromatic C=C bending	-	1406	1405	1405	-
• Fe-O stretching vibration	576	577	579	-	-
• -CH and $-CH_2$ stretching	-	-	2981,2928 & 2862	-	3000 – 2850
• Symmetric stretching and bending vibration of Si-O-Si	-	-	880, 634 & 578	-	1200 – 800 & 670 – 450
• Si-O- C vibration	-	-	1048	-	1050 – 980

.

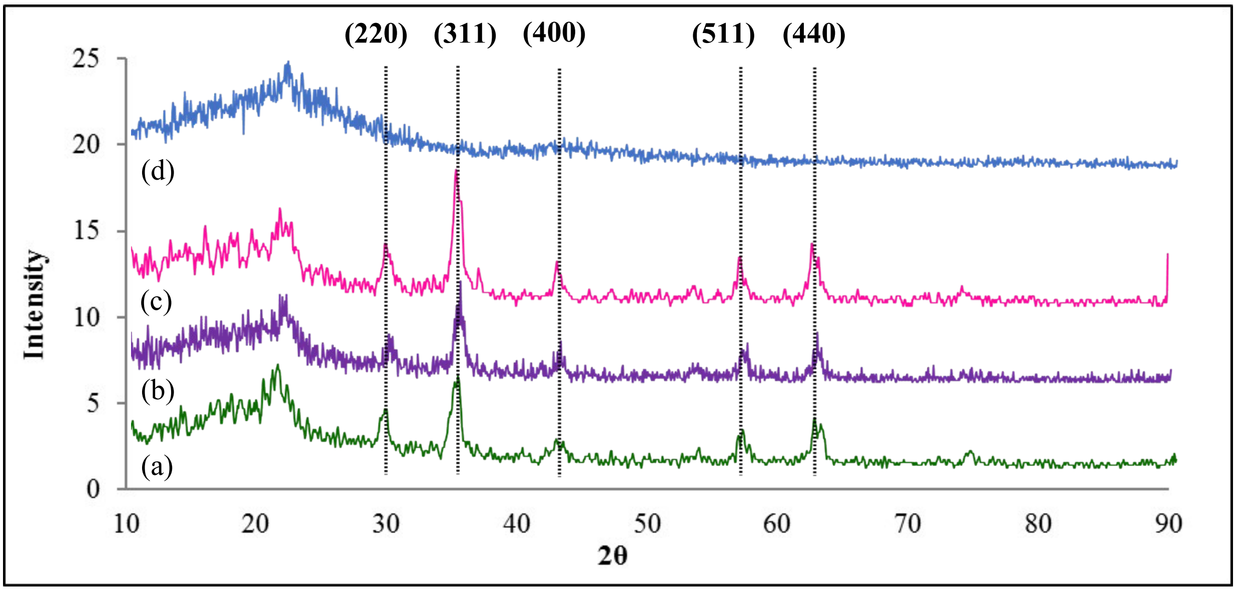

SD 3: XRD spectra of (a) Fe_3O_4 particles, (b) Fe_3O_4-AC, (c) Fe_3O_4@AC-SS and (d) AC.

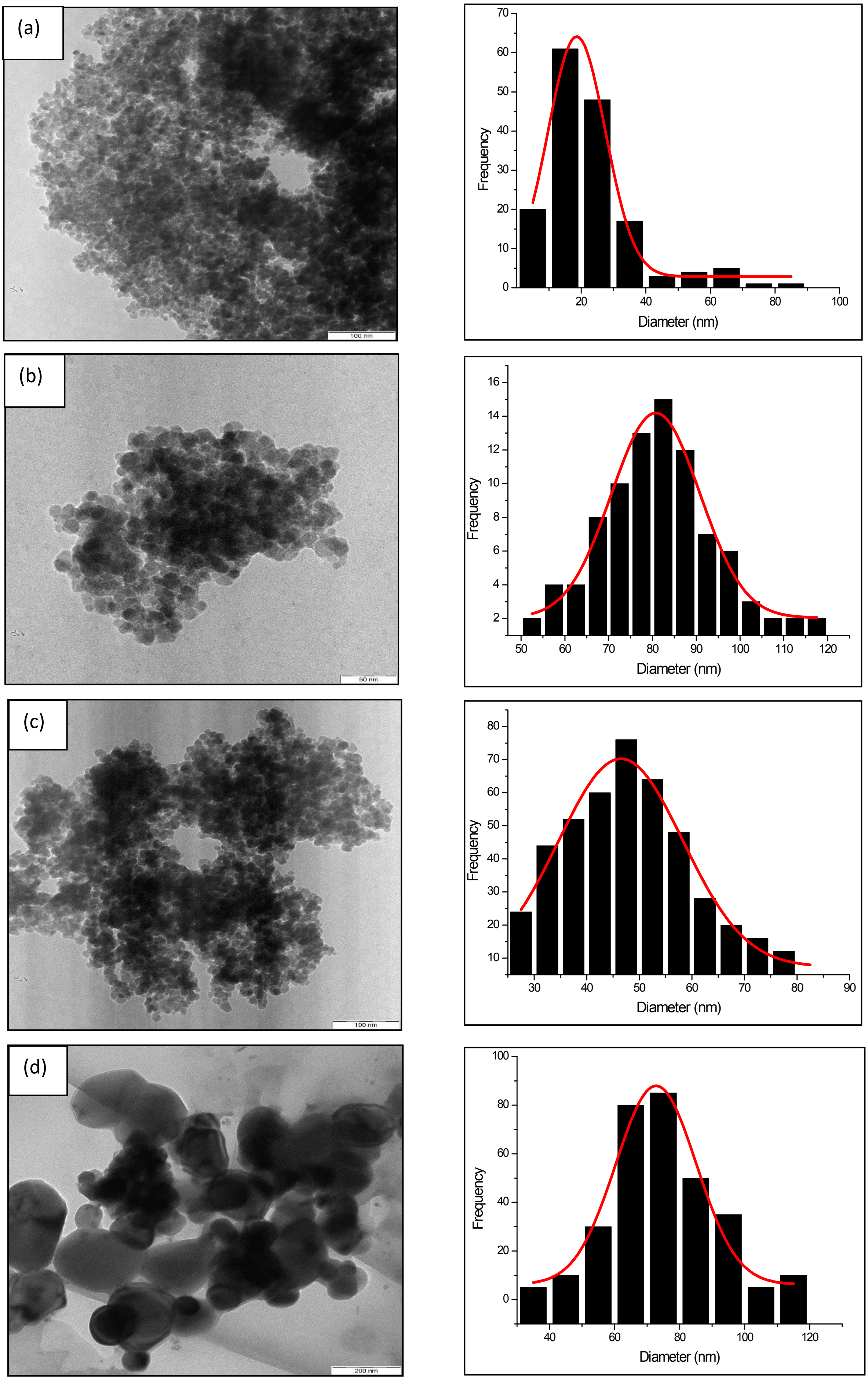

(a)
Frequency
Diameter (nm)
0
10
20
30
40
50
60
70
20
40
60
80
100
(b)
Frequency
Diameter (nm)
2
4
6
8
10
12
14
16
50
60
70
80
90
100
110
120
(c)
Frequency
Diameter (nm)
10
20
30
40
50
60
70
80
30
40
50
60
70
80
90
(d)
Frequency
Diameter (nm)
0
20
40
60
80
100
40
60
80
100
120

SD 4: TEM images of (a) Fe_3O_4 particles, (b) Fe_3O_4@AC, (c) Fe_3O_4@AC-SS, (d) AC and their respective cumulative particle distribution graphs.

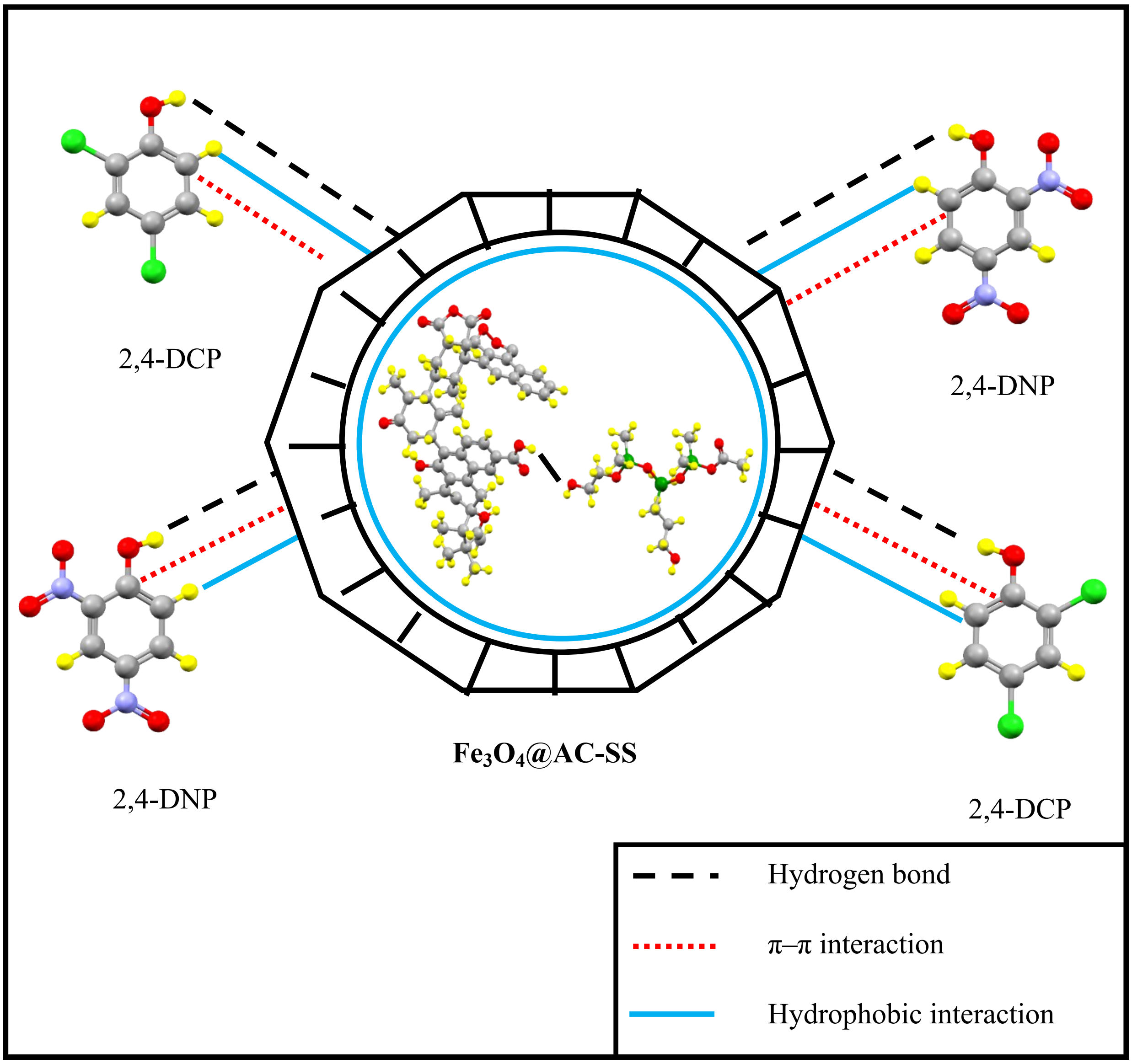

SD 5: Possible proposed adsorption mechanism for 2,4-DCP and 2,4-DNP towards Fe_3O_4@AC-SS.

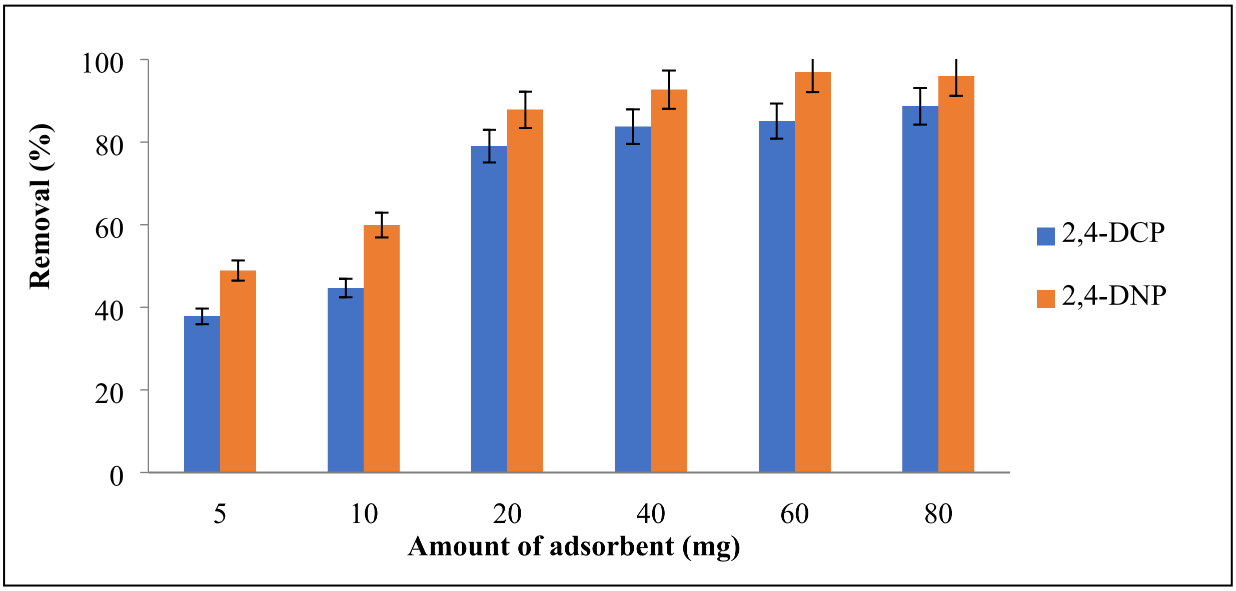

SD 6: Effect of adsorbent dosage on the removal of 2,4-DCP and 2,4-DNP (Condition: pH: 2,4-DCP = 6, 2,4-DNP = 4; contact time: 60 minutes; analyte concentration: 10 mg/L).

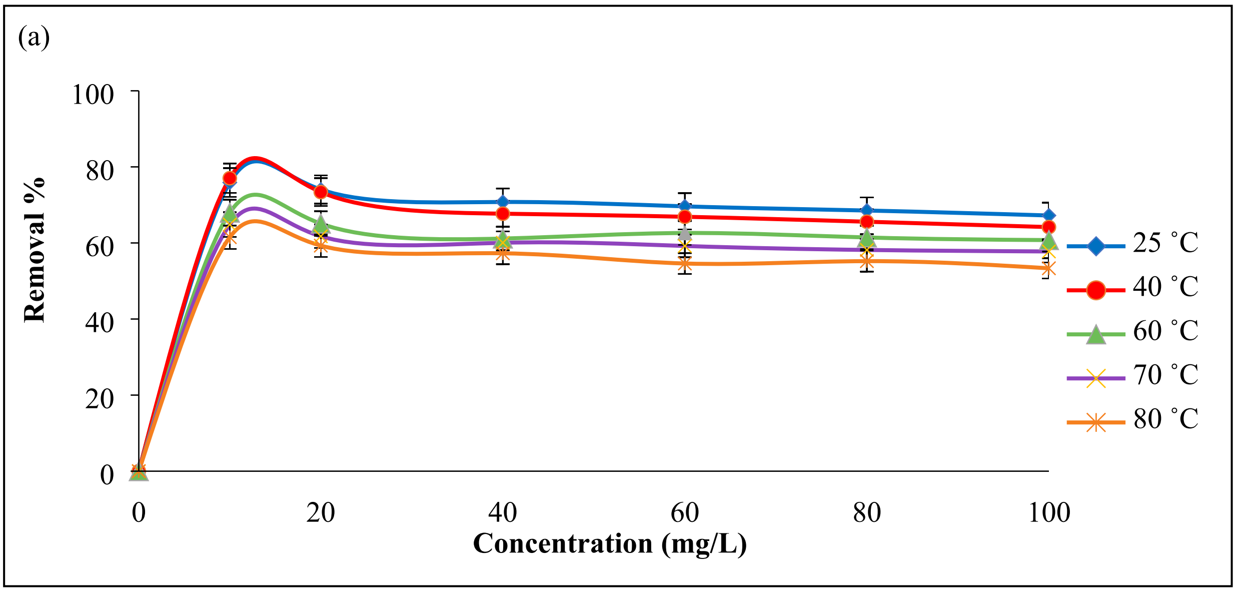

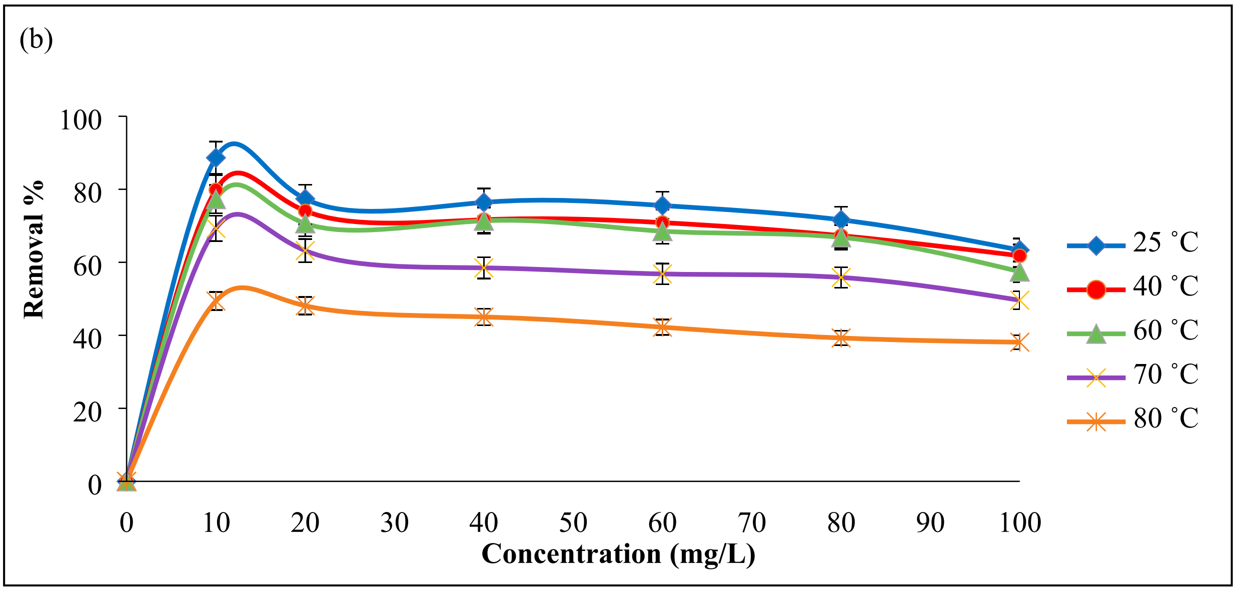

SD 7: Effect of initial concentration on the removal of (a) 2,4-DCP and (b) 2,4-DNP (Condition: pH: 2,4-DCP = 6; 2,4-DNP =4; contact time: 60 minutes; adsorbent dosage: 20 mg).

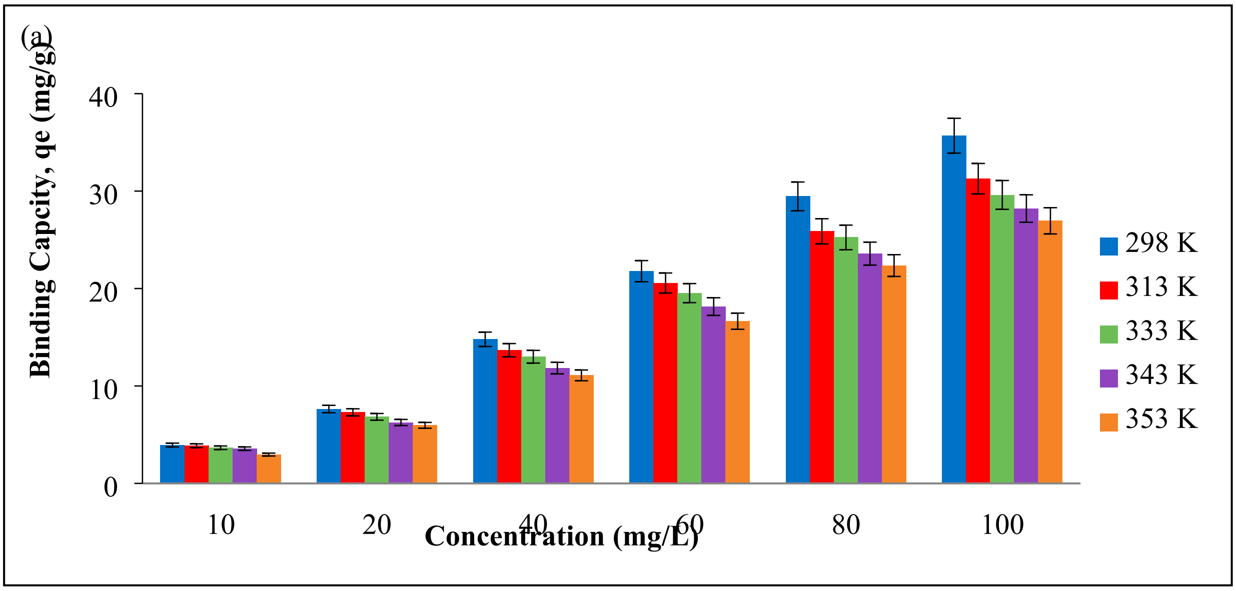

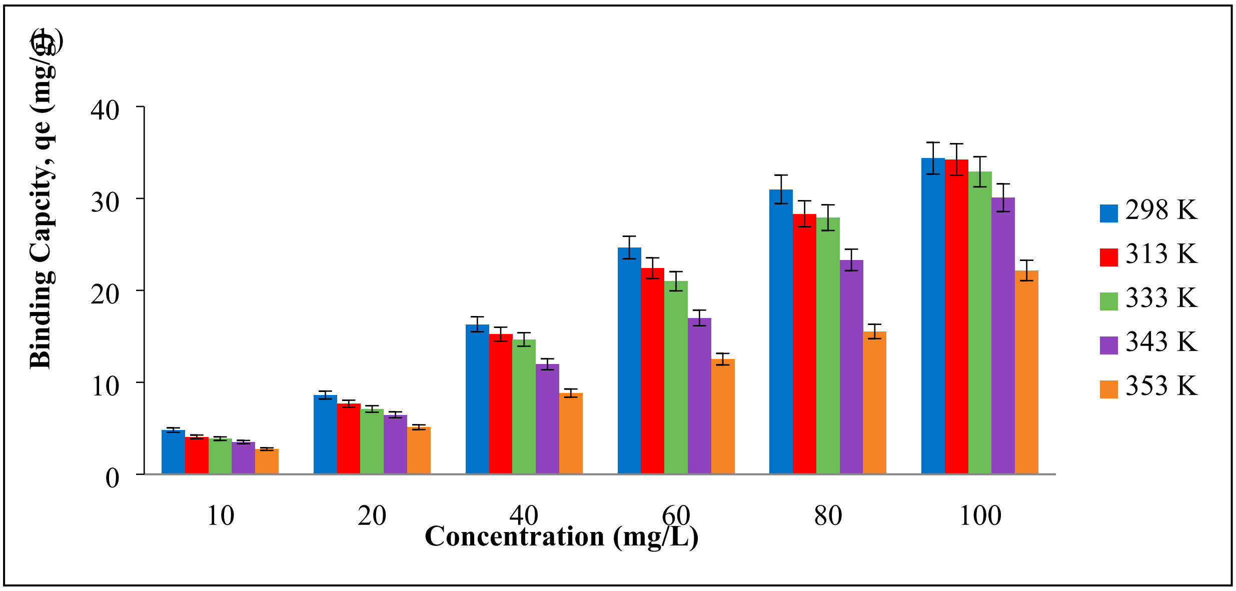

SD 8: Effect of temperature on the removal of (a) 2,4-DCP and (b) 2,4-DNP (Condition: pH: 2,4-DCP = 6; 2,4-DNP = 4; contact time: 60 minutes; adsorbent dosage: 20 mg).

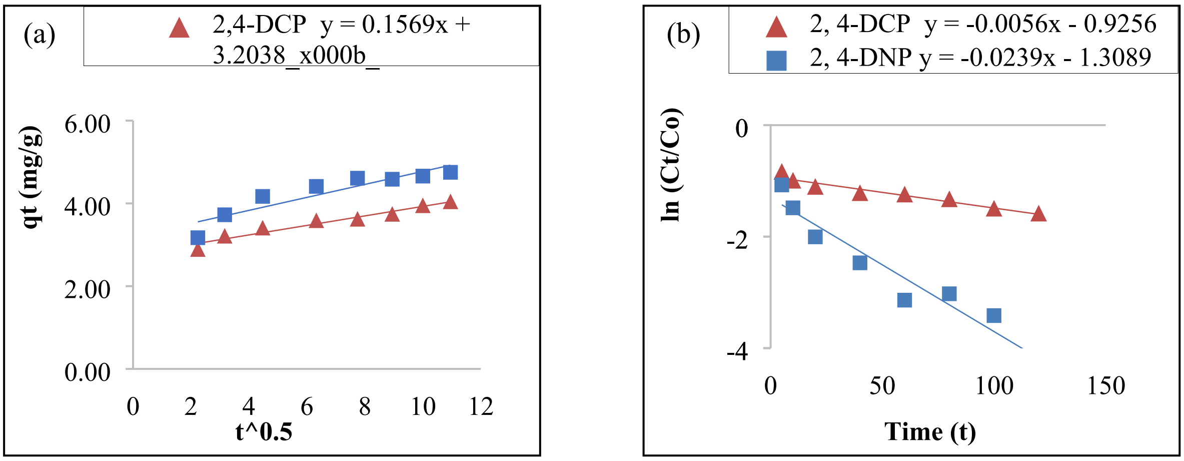

SD 9: (a) Kinetic intraparticle diffusion model and (b) external diffusion model of 2,4-DCP and 2,4-DNP.

SD 10: Details of thermodynamic parameters for the adsorption of 2,4-DCP and 2,4-DNP on Fe_3O_4@AC-SS.

The value of Enthalpy, Entropy, and free Gibbs energy				
Analyte	T (K)	Enthalpy, $\Delta H°$ J/mol	Entropy, $\Delta S°$ J/Kmol	Gibbs energy, $\Delta G°$ kJ/mol
2,4-DCP	298	-9.7091	-30.9630	-337.3522
	313			-25.0985
	333			490.9273
	343			914.1347
	353			1493.0257
2,4-DNP	298	-20.1615	-62.8829	-1078.3492
	313			-509.9129
	333			-234.4607
	343			1194.8560
	353			2954.7957

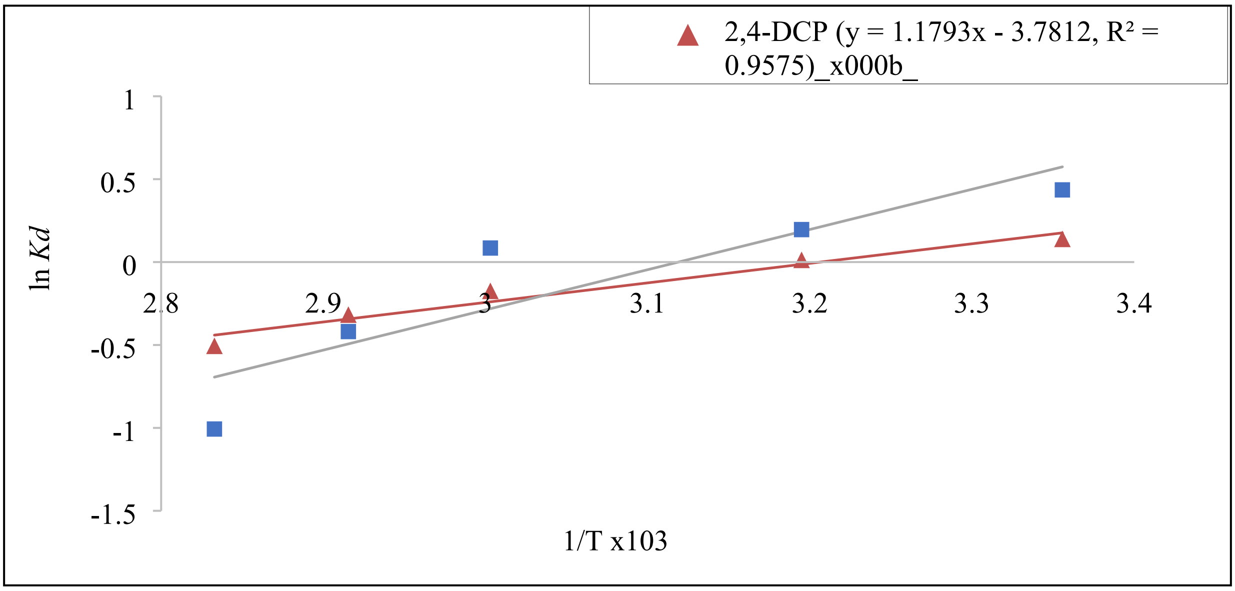

SD 11: Thermodynamic Van't Hoff plot for the adsorption of 2,4-DCP and 2,4-DNP on Fe_3O_4@AC-SS.

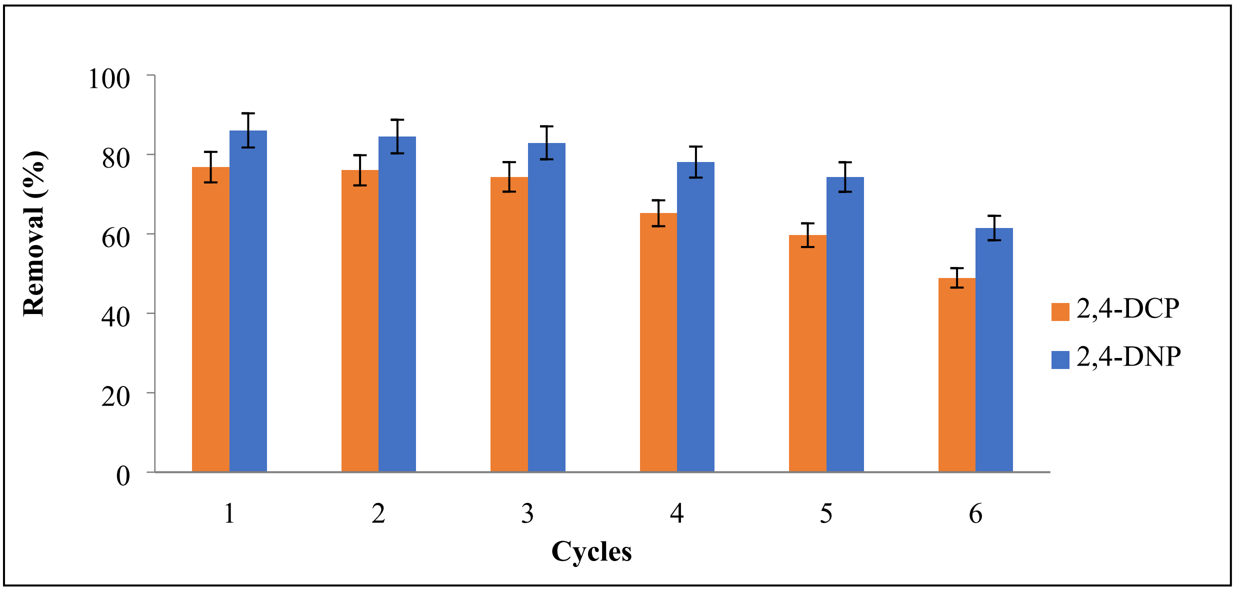

SD 12: Reusability cycles for 2,4-DCP and 2,4-DNP.

SD 13: Validation data on the removal study of 2,4-DCP and 2,4-DNP.

**Analyte**	**Calibration equation**	R^2	**Spiked concentration (mg/L)**	**RSD (%)**		**Recovery (%)**	**RSD of recovery (%)**
				**Inter-day (n=7)**	**Intra-day (n=5)**		
2,4-DCP	y= 1.038703 × 10^{-2}	0.9998	1	2.05	1.65	88.33	1.28
			10	0.65	4.66	79.13	1.29
			40	5.84	5.91	68.13	3.50
2,4-DNP	y= 5.009907 × 10^{-2}	0.9997	1	3.58	3.70	97.22	1.43
			10	2.88	2.46	84.98	1.76
			40	3.91	5.81	78.12	2.20
